# Comprehensive single-cell and bulk transcriptomic analyses to develop an NK cell-derived gene signature for prognostic assessment and precision medicine in breast cancer

**DOI:** 10.3389/fimmu.2024.1460607

**Published:** 2024-10-23

**Authors:** Qianshan Hou, Chunzhen Li, Yuhui Chong, Haofeng Yin, Yuchen Guo, Lanjie Yang, Tianliang Li, Shulei Yin

**Affiliations:** ^1^ National Key Laboratory of Immunity & Inflammation, Institute of Immunology, Naval Medical University, Shanghai, China; ^2^ School of Pharmacy, Naval Medical University, Shanghai, China

**Keywords:** breast cancer, natural killer (NK) cell, scRNA-seq, prognostic signature, tumor microenvironment, immunotherapy

## Abstract

**Background:**

Natural killer (NK) cells play crucial roles in mediating anti-cancer activity in breast cancer (BRCA). However, the potential of NK cell-related molecules in predicting BRCA outcomes and guiding personalized therapy remains largely unexplored. This study focused on developing a prognostic and therapeutic prediction model for BRCA by incorporating NK cell-related genes.

**Methods:**

The data analyzed primarily originated from the TCGA and GEO databases. The prognostic role of NK cells was evaluated, and marker genes of NK cells were identified via single-cell analysis. Module genes closely associated with immunotherapy resistance were identified by bulk transcriptome-based weighted correlation network analysis (WGCNA). Following taking intersection and LASSO regression, NK-related genes (NKRGs) relevant to BRCA prognosis were screened, and the NK-related prognostic signature was subsequently constructed. Analyses were further expanded to clinicopathological relevance, GSEA, tumor microenvironment (TME) analysis, immune function, immunotherapy responsiveness, and chemotherapeutics. Key NKRGs were screened by machine learning and validated by spatial transcriptomics (ST) and immunohistochemistry (IHC).

**Results:**

Tumor-infiltrating NK cells are a favorable prognostic factor in BRCA. By combining scRNA-seq and bulk transcriptomic analyses, we identified 7 NK-related prognostic NKRGs (CCL5, EFHD2, KLRB1, C1S, SOCS3, IRF1, and CCND2) and developed an NK-related risk scoring (NKRS) system. The prognostic reliability of NKRS was verified through survival and clinical relevance analyses across multiple cohorts. NKRS also demonstrated robust predictive power in various aspects, including TME landscape, immune functions, immunotherapy responses, and chemotherapeutic sensitivity. Additionally, KLRB1 and CCND2 emerged as key prognostic NKRGs identified through machine learning and external validation, with their expression correlation with NK cells confirmed in BRCA specimens by ST and IHC.

**Conclusions:**

We developed a novel NK-related gene signature that has proven valuable for evaluating prognosis and treatment response in BRCA, expecting to advance precision medicine of BRCA.

## 1 Introduction

Breast cancer (BRCA), known as “the first killer of women”, has been attracting more attention globally due to its high incidence rate and large patient population (1, 2). In 2022, BRCA accounted for 11.6% of all newly diagnosed cancer cases worldwide, ranking second only to lung cancer (3). The death toll reached 665,684 and continues to rise at an annual rate of approximately 0.6% (4).

Consequently, the early diagnosis and treatment of BRCA have emerged as a principal research focus in recent years. Despite significant advancements in molecular oncology and therapeutics that have substantially improved the five-year survival rate of BRCA patients, certain individuals still experience poor outcomes due to factors including delayed diagnosis, therapeutic resistance, metastasis, etc. (5). Chemotherapy, endocrine therapy, targeted therapy, and immunotherapy have become the common treatments for BRCA (6–10). Notably, immunotherapy, particularly immune checkpoint blockade (ICB), has achieved significant advancements, with a 482.1% increase in related research over the past decade (11). Despite these advancements, the current unsatisfactory response rates to immunotherapy remain a critical issue, hindering further improvements in BRCA survival rates (9, 12). Only 5% of patients, mainly those with triple-negative breast cancer (TNBC), experience a sustained response (13, 14). Consequently, there is an urgent need to identify new molecular targets to enhance BRCA treatment and to perform individualized assessments of therapeutic response and prognosis to broadly increase survival rates.

The tumor microenvironment (TME) is a complex and multifaceted system, predominantly consisting of tumor cells, surrounding immune cells, fibroblasts, interstitial tissues, capillaries, and a variety of cytokines and chemokines (15). The TME is closely linked to tumor growth, influencing all stages of tumor development, including initiation, invasion, and malignant transformation (16). Within the TME, alongside the well-documented T cells known for their tumoricidal capabilities, NK cells also play a critical anti-cancer immune effect. As a subclass of innate immune cells, NK cells possess functions such as immune surveillance (17), pathogen elimination (18), and anti-aging (19), with particularly potent tumor-killing capabilities (20, 21). They eliminate tumor cells through three main manners: direct cytotoxicity, secretion of cytokines and chemokines to stimulate further immune responses, and collaboration with antibodies on the cell surface to foster an adaptive immune response (22). Recent studies have demonstrated that NK cells could augment their anti-tumor efficacy against glioblastoma via epigenetic reprogramming (23). Furthermore, NK cells can be activated by decreasing glycocalyx thickness, thereby inhibiting the metastasis of BRCA (24). Thus, NK cells can greatly impact the effectiveness of immunotherapy, and new immunotherapies leveraging NK cells as a foundation are currently highly favored (25, 26). Considering the essential role of NK cells in the immune system and tumor immunity, a comprehensive characterization of NK-related molecules is critical for uncovering novel biomarkers associated with BRCA diagnosis and treatment efficacy. Nonetheless, current research in this field is still relatively scarce.

Single-cell sequencing (scRNA-seq) is a cutting-edge technology that enables the extraction, amplification, and high-throughput analysis of genomes or transcriptomes at the single-cell level (27). Advances in scRNA-seq have significantly improved our understanding of the immune system, thereby facilitating and expediting both basic research and clinical applications in cancer (28). Therefore, we believe that scRNA-seq can also be employed to uncover and develop novel NK-related biomarkers relevant to tumor prognosis and immunotherapy, enhancing prognostic prediction for BRCA.

**Figure 1:**
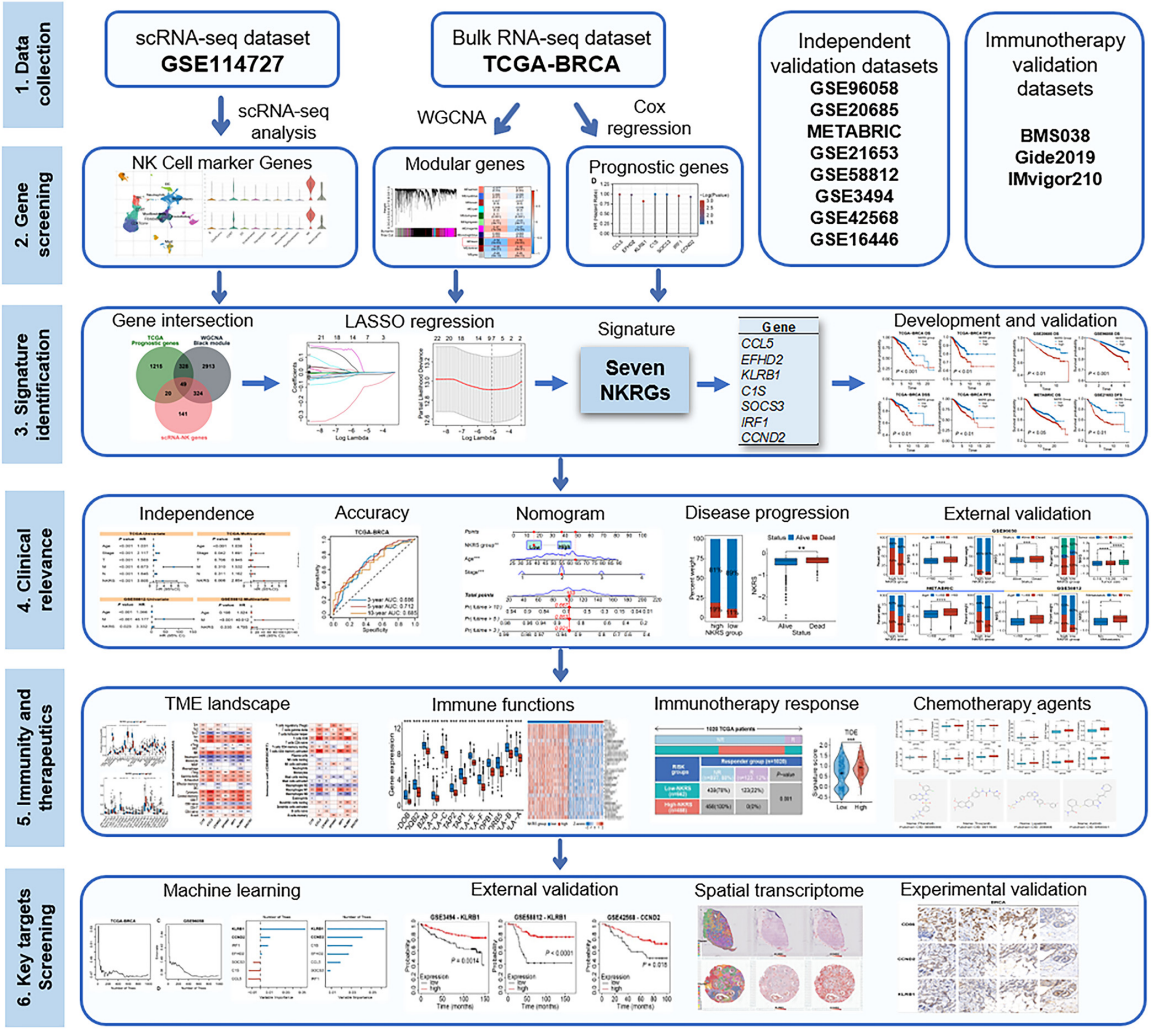
Flowchart of the present study.

In this study, through integrating single-cell and bulk transcriptomic analyses, we have developed a robust prognostic signature comprising seven NK-related molecules for BRCA. Moreover, the predictive capacity of the signature has been validated in multiple cohorts, and it has been utilized to assess TME landscapes, anti-tumor immunity, and therapeutic preferences. Furthermore, we identified key prognostic NKRGs and experimentally confirmed their expression patterns in BRCA tissues. The workflow of this study is depicted in **Figure 1**.

## 2 Materials and methods

### 2.1 Data acquisition

The Cancer Genome Atlas (TCGA, https://portal.gdc.cancer.gov) database was utilized to extract bulk RNA-seq data and clinical information of 1109 BRCA samples. Additionally, scRNA-seq profiles of BRCA (GSE114727) consisting of 19,676 cells were analyzed using the TISCH (Tumor Immune Single-cell Hub) database (http://tisch1.comp-genomics.org/). External validation of the prognostic signature and clinical relevance analysis was conducted using five independent BRCA cohorts: GSE20685 (n=327), GSE96058 (n=3273), METABRIC (n=1897), GSE21653 (n=241), and GSE58812 (n=107, TNBC). Moreover, independent GEO cohorts, including GSE3494 (n=236), GSE42568 (n=104), and GSE16446 (n=107), were employed to elucidate the prognostic role of key NKRGs. Three patient cohorts (BMS038, Gide2019, and IMvigor210) undergoing ICB treatment were introduced to evaluate the potential of this NKRS in predicting patient sensitivity to immunotherapy (29–32).

### 2.2 Investigation of NK cells abundance and stratified survival analysis

The MCPcounter algorithm was employed to quantify the abundance of tumor-infiltrating NK cells based on bulk transcriptomic data (33). To clarify the prognostic role of NK cells, grouping and survival analyses were performed based on the abundance of tumor-infiltrating NK cells. With the use of “survminer” and “survival” packages, we determined the optimal cutoff value for survival analysis. Subsequently, Kaplan–Meier curves were generated to illustrate the survival differences.

### 2.3 Acquisition of NK-related genes affecting ICB resistance by comprehensive single-cell analysis and bulk WGCNA

Utilizing the online platform TISCH, we conducted standard scRNA-seq analysis on the dataset GSE114727, encompassing single-cell sequencing data of 19,676 immune cells from 8 patients diagnosed with primary BRCA. This comprehensive process, comprising data preconditioning and quality control, cell clustering and annotation, and differential gene analysis and visualization, was performed in accordance with the official operating instructions on the TISCH website. NK-related genes (NKRGs) were initially obtained by differential gene analysis.

To discover novel NKRGs associated with ICB resistance, gene set variation analysis (GSVA) and WGCNA were performed. The GSVA based on the “IOBR” package, a practical tool to quantify tumor immunity-related indicators, including ICB resistance, was conducted to obtain the ICB resistance score for each sample (34, 35). WGCNA was then employed to select the modules and genes with the highest correlation to ICB resistance. After performing the sample clustering and outlier removal, the rational soft threshold was determined for optimal running efficiency and stability. Module genes with the highest and most statistically significant correlation with ICB resistance will be subject to be taken to intersect with NKRGs, and derived candidates will be further analyzed for prognostic screening.

### 2.4 Generation and validation of the NK-related prognostic signature

Prognostic gene screening was accomplished using univariate Cox regression analysis, and genes with P-values less than 0.05 were retained and intersected with the two aforementioned groups of candidates to yield NKRGs with potential impact on BRCA prognosis and immunotherapy resistance. Subsequently, least absolute shrinkage and selection operator (LASSO) regression was then carried out to determine the ideal NKRGs and their coefficients for comprising the signature (23). Referring to previously described methods, each patient was assigned an NK-related risk score (NKRS) (36, 37). Based on the optimal cutoff NKRS, patients in the respective cohort could be stratified into a high-NKRS group and a low-NKRS group. The formula for calculating NKRS is abbreviated as:


$$\text{NKRS}=\sum_{i=1}^{n}\left[\text{coefficient }\left(\text{NKRGi}\right)\;*\text{ expression }\left(\text{NKRGi}\right)\right]$$

The survival disparity between NKRS groups was exhibited using the aforementioned packages for survival analysis. Furthermore, to bolster the credibility of the NKRS, multiple types of survival, as well as external validation cohorts, were also incorporated into the NKRS system, utilizing a similar approach as described above.

### 2.5 Evaluating the clinicopathologic significance of NKRS

To assess the precision of NKRS in forecasting BRCA patient survival, we generated time-dependent receiver operating characteristic (ROC) curves using the “timeROC” package (38). We further investigated the relationship between NKRS and various clinicopathological factors, such as age, stage, and metastasis, by comparing these parameters across the two NKRS groups. Univariate and multivariate Cox regression analyses were conducted to determine if NKRS and clinicopathological factors can serve as independent predictors of prognosis in different BRCA cohorts. Subsequently, we constructed a predictive nomogram with the use of “rms” and “regplot” packages to enable the accurate assessment of patient survival (37).

### 2.6 Gene set enrichment analysis

Initially, we utilized the R package “Limma” to identify differentially expressed genes and fold change among different NKRS groups. Subsequently, we employed the “org.Hs.eg.db” and “clusterProfiler” packages to conduct the GSEA, aiming to elucidate the disparities of functional enrichment and biological pathways between high-NKRS and low-NKRS tumors (39). Gene set files of Gene Ontology (GO) terms, Kyoto Encyclopedia of Genes and Genomes (KEGG) pathways and HALLMARK pathways in gmt format for GSEA were obtained from the MsigDB (40).

### 2.7 Tumor microenvironment analysis

To evaluate the immunological relevance of the NKRS, we assessed the abundance of tumor-infiltrating immune cells (TICs) and computed microenvironmental scores. Specifically, we leveraged the CIBERSORT and ImmunecellAI algorithms to quantify the intratumoral immune cell content (41, 42). The CIBERSORT analysis adhered to the official recommendations and was replicated 1000 times for robustness. For ImmunecellAI, we utilized its online immune cell abundance analysis function following the official guidelines. Furthermore, the ESTIMATE algorithm allowed us to assess immune scores, stromal scores, ESTIMATE scores, and tumor purity (43). To examine the association between NKRG expression and TIC infiltration, we employed the Spearman correlation analysis and generated correlation heat maps.

### 2.8 Characterization of immune function and immunotherapy vulnerability

To comprehensively reveal the value of this NKRS in profiling the immune function and sensitivity to ICB therapy of patients, we performed the GSVA analysis based on immune gene sets using the IOBR package to characterize the immune function of tumors in different NKRS groups (44). The results were visualized via heat maps and box plots. To further explore the clinical significance of NKRS in cancer immunotherapy, we predicted the response of high- and low-NKRS groups to immunotherapy using the Tumor Immune Dysfunction and Exclusion (TIDE) algorithm and the Immunophenoscore (IPS) (45, 46). Additionally, transcriptomic and survival analyses were conducted in several real-world cohorts undergoing ICB therapy to validate our predictive findings.

### 2.9 Screening of candidate chemotherapeutic drugs

Utilizing the “oncoPredict” package, we evaluated the half-maximal inhibitory concentration (IC50) of common clinical chemotherapeutic and targeted drugs, including paclitaxel, platinum, fluorouracil, PARP inhibitors, etc., to further analyze the chemosensitivity of tumors (47). Additionally, we analyzed the transcriptomic differences between these two NKRS groups and submitted them to the Connectivity Map (Cmap, https://clue.io/) platform to identify potential agents for therapeutic intervention of high-NKRS BRCA cases (48). Structural information on those compounds was obtained from PubChem.

### 2.10 Screening and validation of key prognostic NKRGs

A machine-learning-based framework was applied to screen key NKRGs affecting BRCA prognosis. Specifically, we assessed and visualized the significance of signature NKRGs in influencing the prognosis of BRCA using a random forest algorithm based on the “randomForestSRC” package and cross-validated them in different cohorts (49). The diagnostic ROC curve and differential expression profiles were also employed to further identify key NKRGs, whose effect on patient survival was validated in multiple external BRCA cohorts.

### 2.11 Experimental verification of the expression pattern of key NKRGs in BRCA

To characterize the expression of key NKRGs and their NK cell relevance in BRCA, immunohistochemical (IHC) experiments on BRCA specimens were performed. Tissue microarrays containing 40 cases of BRCA tissues were purchased from Servicebio (Wuhan, China). IHC procedures were conducted in accordance with previous publications (36, 37). Primary antibodies for IHC staining against KLRB1 (57537-1-lg), CCND2 (10934-1-AP), and CD56 (14255-1-AP) were purchased from Proteintech (Wuhan, China).

### 2.12 Statistical analysis

For statistical analysis, R software (version 4.1.2) along with various online tools such as TISCH, ImmunecellAI, and Cmap, were implemented in this study. Our study employed several R packages such as “WGCNA”, “limma”, “ggplot2”, “survival”, “IOBR”, “GSVA”, and “oncoPredict”, and their specific applications are detailed in the respective sections. To compare continuous variables, we relied on the Student’s t-test, while the chi-square test was used for categorical variables. Additionally, the Wilcoxon test was employed to compare gene expression across groups. A p-value less than 0.05 was considered statistically significant.

## 3 Results

### 3.1 A higher abundance of tumor-infiltrating NK cells indicated a better prognosis for BRCA

**Figure 2:**
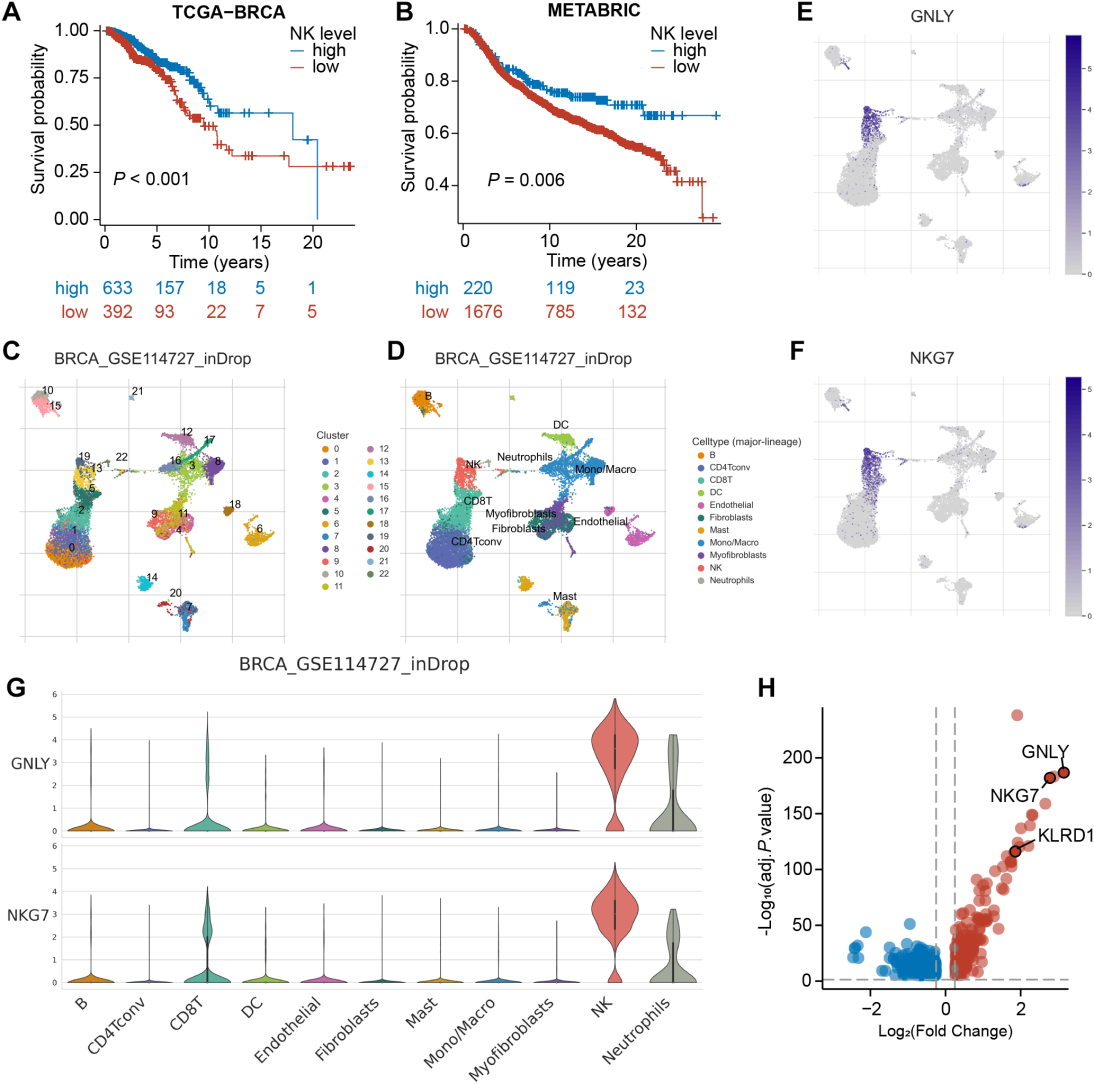
Identification of NK marker genes via scRNA-seq analysis. **(A, B)** Kaplan–Meier analyses revealed a significant disparity between the high-NK and low-NK groups in the TCGA-BRCA and METABRIC cohorts. **(C)** The UMAP depicts 22 immune cell clusters in the GSE114727 dataset. **(D)** The UMAP depicts 11 identified cell types. **(E, F)** Feature plots exhibit differential expression of GNLY and NKG7 across various cell clusters. **(G)** Violin plots demonstrate that GNLY and NKG7 were predominantly expressed in NK cells. **(H)** A volcano plot shows the differential expression of NK cell-related gene, with up-regulated genes in red and down-regulated genes in blue.

To confirm the prognostic significance of NK cells in BRCA, we initially utilized the MCPcounter algorithm to estimate the abundance of tumor-infiltrating NK cells in bulk RNA-seq samples. Subsequently, we categorized the TCGA-BRCA samples into high- and low-NK groups (**Supplementary Table S1**). Kaplan–Meier analysis revealed a significant difference between these two groups (P<0.001), with the high-NK group exhibiting longer survival (**Figure 2A**). This finding suggests that NK cells have a favorable prognostic impact on BRCA. Consistently, this trend was also observed in the METABRIC cohort (P=0.006, **Figure 2B**; **Supplementary Table S1**), underscoring the critical role of NK cells in influencing BRCA prognosis.

### 3.2 Single-cell transcriptomic analysis identified 534 NKRGs

Subsequently, we processed the scRNA-seq dataset GSE114727 through the TISCH platform and arranged 19,676 cells into 22 clusters, which were further classified into 11 cell types through annotation, comprising B cells, CD8^+^ T cells, NK cells, fibroblasts, and so on (**Figures 2C, D**). As demonstrated in UMAPs, cells in cluster 19 and cluster 13 were identified as NK cells. The expression of typical marker genes of NK cells was depicted in UMAPs and violin plots (**Figures 2E-G**; **Supplementary Figures S1A, B**). Furthermore, the volcano plot revealed 534 NK-related genes (NKRGs) identified by differential analysis between cell types, which were subject to subsequent screening (**Figure 2H**).

### 3.3 Bulk transcriptome-based WGCNA screened module genes associated with ICB resistance

**Figure 3:**
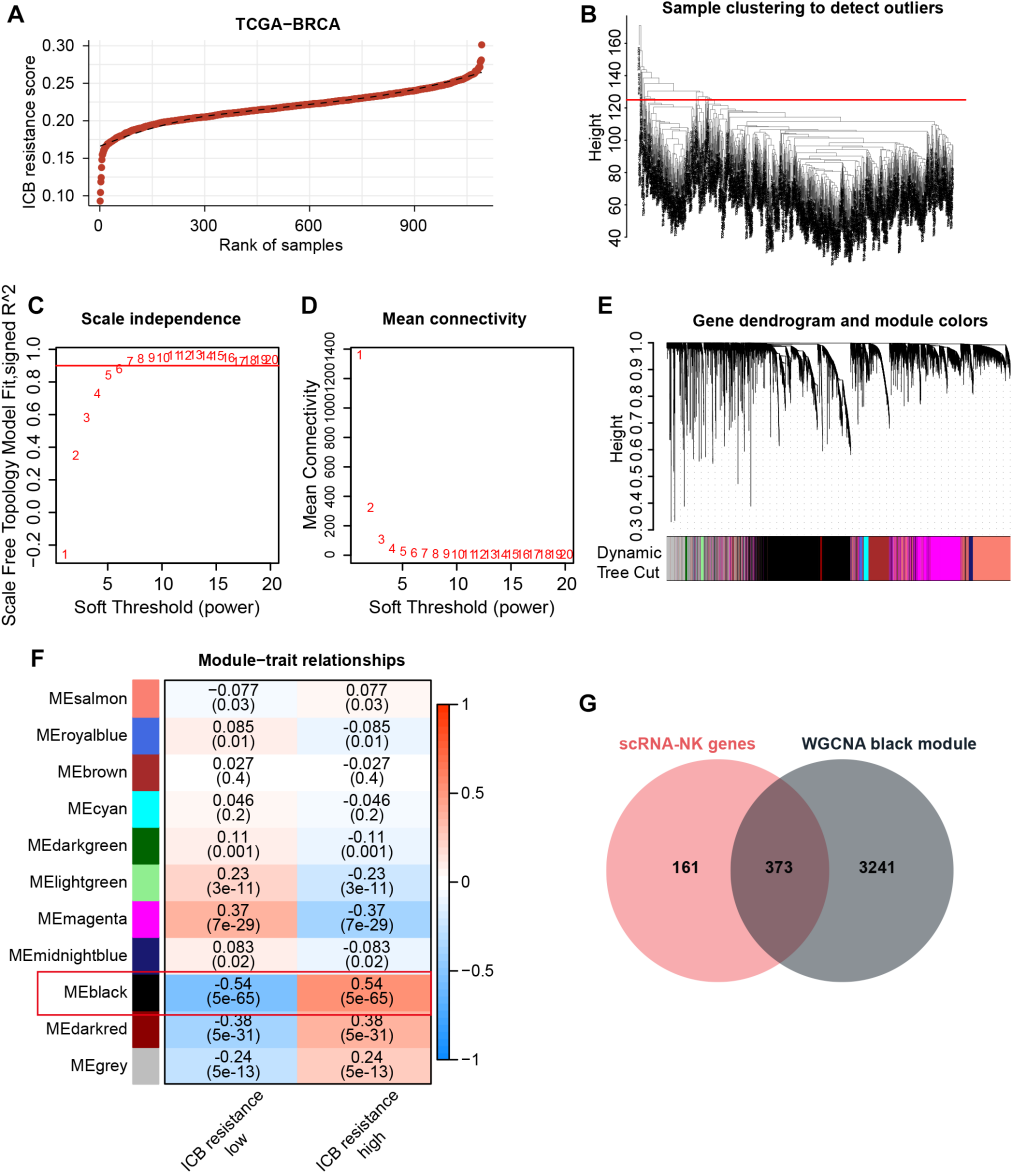
Identification of ICB resistance-related modules through WGCNA. **(A)** ICB resistance scores of samples in the TCGA-BRCA cohort. **(B)** The outlier samples were removed from the analysis. **(C, D)** The optimal soft-thresholding power was determined to be 6. **(E)** A dynamic tree cut was visualized. **(F)** Correlation analysis between modules and traits. **(G)** The Venn diagram demonstrates the intersection of NK marker genes and ICB resistance-related genes.

Since NK cells can modulate anti-tumor immunity and thus affect immunotherapeutic efficacy through a variety of mechanisms, including direct killing and indirect action, our study further hopes to obtain NK-related targets tightly associated with immunotherapeutic efficacy. Therefore, with the use of GSVA, we assigned ICB resistance scores to samples in the TCGA-BRCA cohort (**Figure 3A**). Thereafter, WGCNA was conducted to acquire ICB resistance-related modules. First, outlier samples were excluded to ensure the sample quality (**Figure 3B**), and the optimal soft thresholding value was determined to be 6 (**Figures 3C, D**). Subsequently, a cluster dendrogram based on gene correlation was constructed, and the preliminary genetic modules were obtained (**Supplementary Figures S1C, D**). After merging several minor modules, we identified a total of 11 modules for analysis (**Figures 3E, F**). Within these modules, the black module possessed the strongest correlation with ICB resistance scores (**Figure 3F**). By intersecting the black-module genes with NKRGs, 373 ICB resistance-related NKRGs were obtained (**Figure 3G**).

### 3.4 An NK-related signature consisting of 7 prognostic NKRGs was developed

**Figure 4:**
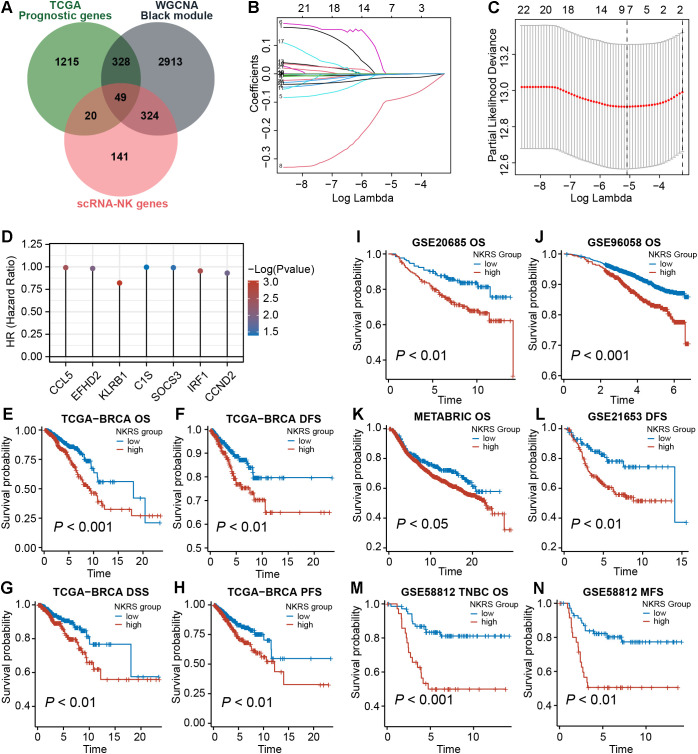
Establishment of the NK-related risk signature. **(A)** Taking intersections to obtain prognostic NKRGs associated with ICB resistance. **(B, C)** LASSO regression analysis identified signature genes. **(D)** The hazard ratio (HR) of each signature NKRG. **(E-N)** Multiple types of survival analyses were conducted on TCGA datasets as well as validation datasets including GSE20685, GSE96058, GSE21653, GSE58812 and METABRIC cohort. Different survival types included in this figure: OS (overall survival), DFS (disease-free survival), PFS (progression-free survival), DSS (disease-specific survival), MFS (metastasis-free survival).

To improve the prognostic accuracy of the signature, we identified 1612 prognostic genes by univariate Cox regression in the TCGA-BRCA cohort. Further, we intersected them with candidate genes obtained from the single-cell and WGCNA analyses described above, yielding 49 promising NKRGs (**Figure 4A**). Through LASSO regression analysis, 7 prognostic NKRGs were selected to develop the signature (**Figures 4B, C**). The NK-related risk score (NKRS) of a sample could be generated using the expression and coefficients of these 7 NKRGs (**Supplementary Table S2**). The hazard ratio (HR) of each signature NKRG is shown in **Figure 4D**.

Aiming to evaluate the prognostic significance of this signature, we calculated the NKRS of each sample in the TCGA-BRCA cohort and conducted survival analyses after grouping samples according to the optimal cutoff. As observed from **Figures 4E-H**, the low-NKRS group exhibited prolonged overall survival (OS), disease-free survival (DFS), disease-specific survival (DSS), and progression-free survival (PFS). Moreover, the improved prognosis in low-NKRS patients was also confirmed in several validation cohorts, including GSE20685, GSE96058, METABRIC, GSE21653, and GSE58812 (**Figures 4I-N**). Notably, in the TNBC cohort GSE58812, NKRS was capable of suggesting not only OS but also metastasis-free survival (MFS), demonstrating the superior performance of the NKRS as a prognostic indicator for BRCA (**Figures 4M, N**).

### 3.5 The independent prognostic role of the NKRS was determined in multiple cohorts

**Figure 5:**
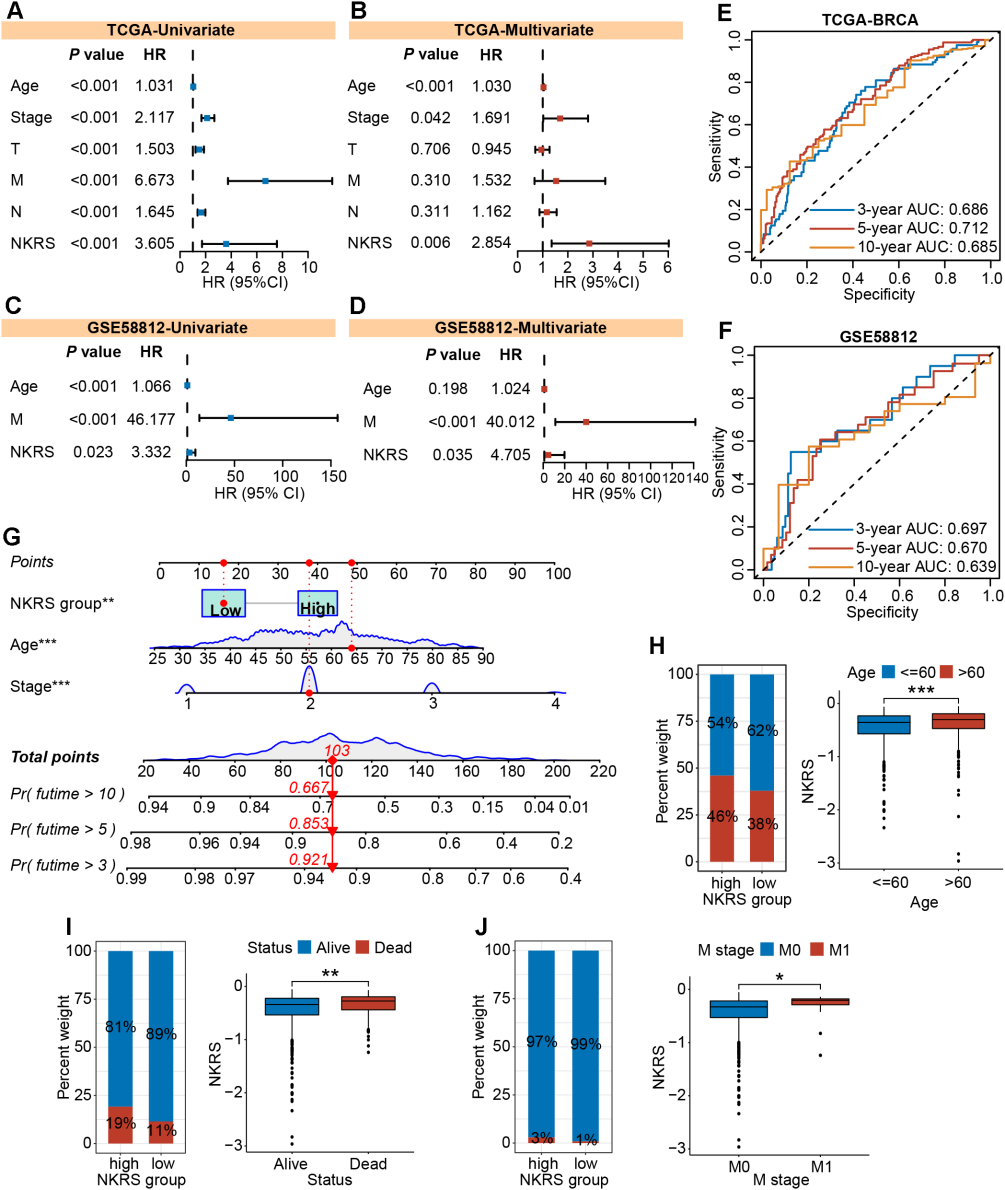
The independent prognostic role and clinical relevance of NKRS. **(A-D)** The prognostic independence of NKRS was assessed in TCGA-BRCA and GSE58812 cohorts. **(E, F)** The time-dependent ROC curves of NKRS in TCGA-BRCA and GSE58812 cohorts. **(G)** A predictive nomogram model was established. **(H-J)** The histograms and box plots illustrated that the high-NKRS group was correlated with advanced age, increased mortality, and a higher incidence of tumor metastasis. In this figure, T, N, M are clinical indicators derived from the TNM staging system. “T” represents tumor, “N” represents lymph node, and “M” represents metastasis (*P<0.05, **P<0.01, ***P<0.001).

To investigate whether NKRS could serve as an independent prognostic factor for BRCA, we carried out univariate and multivariate Cox regression analyses and found that the influence of NKRS on BRCA progression would not vary with other clinical factors. This indicates its independent prognostic role (**Figures 5A, B**). Similar analyses conducted in validation cohorts also strongly supported this finding (**Figures 5C, D**; **Supplementary Figure S2A**). Time-dependent ROC curves were plotted to assess the reliability of the NKRS. The areas under the curve at different time points indicated that this NKRS carries relatively good prognostic accuracy in both the TCGA-BRCA and validation cohorts (**Figures 5E, F**). Integrating independent prognostic indicators (stage, age, and NKRS), a predictive nomogram was created for quantitatively analyzing patient survival, further improving the clinical applicability of NKRS (**Figure 5G**).

### 3.6 The NKRS exhibited favorable clinicopathological relevance

The NKRS was strongly associated with BRCA prognosis, whereas its correlation with clinicopathologic indicators needed to be further evaluated. Thereafter, histograms and box plots demonstrated that the high-NKRS group was more prevalent among patients older than 60 and prone to death (**Figures 5H, I**). As expected, a higher proportion of patients with metastatic cancer was found in the high-NKRS group (**Figure 5J**). In the validation sets (GSE96058, MATABRIC, GSE58812), similarly, it was observed that patient age, survival status, tumor size, and metastasis status were all closely related to NKRS (**Supplementary Figure S2B**). The above results suggest that NKRS not only correlates with the survival of BRCA patients, but also shows good relevance to clinicopathological parameters such as tumor size and metastasis, making it a promising prognostic indicator.

### 3.7 Immune pathways functioned actively in low-NKRS tumors

**Figure 6:**
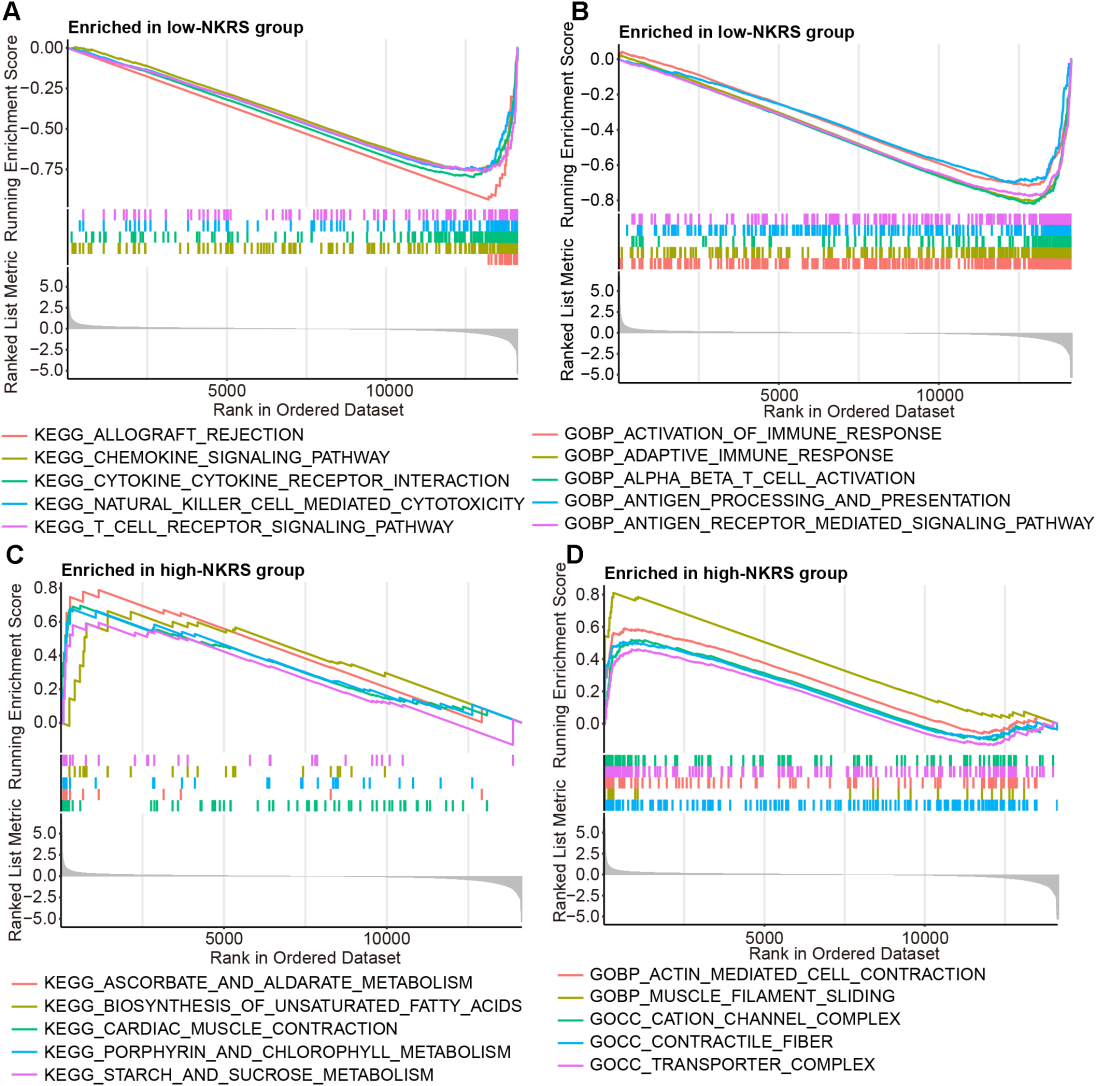
Discovering the association between NKRS and cancer immunity. **(A, B)** GSEA revealed specific immunity-related signaling pathways were enriched in the low-NKRS group. **(C, D)** GSEA indicated that the high-NKRS group was prominently enriched in certain pathways.

The reasons and mechanisms behind the significant effect of NKRS on BRCA prognosis were yet to be clarified. GSEA was conducted between NKRS groups, revealing that certain immunity-related signaling pathways, including chemokine signaling, natural killer cell-mediated cytotoxicity, T cell receptor signaling, activation of immune response, and inflammatory response pathway were enriched significantly in low-NKRS group (**Figures 6A, B**; **Supplementary Figure S3B**), whereas biosynthesis of unsaturated fatty acid, transporter complex and pancreas beta cells were closely associated with high-NKRS group (**Figures 6C, D**; **Supplementary Figure S3A**). These analysis results suggested that the improvement of BRCA prognosis in the low-NKRS group may be generated by immune responses and the tumor immune microenvironment (TME).

### 3.8 Significant differences in TME between NKRS-based risk groups

**Figure 7:**
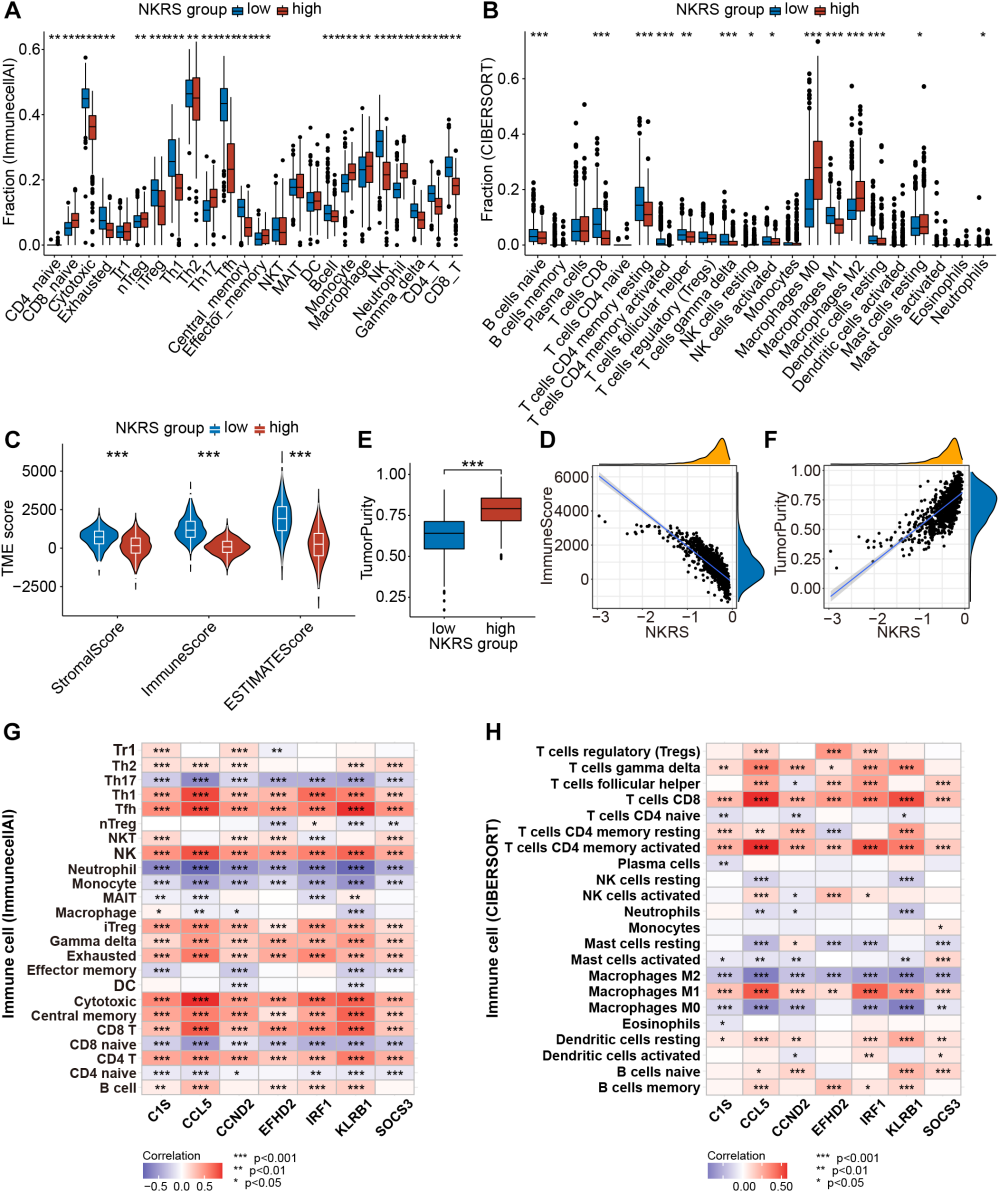
Analysis of the TME landscape. **(A, B)** ImmunecellAI and CIBERSORT algorithms were utilized to calculate the proportions of TICs. **(C)** The violin plot shows the differences in stromal score, immune score, and ESTIMATE score between the low- and high-NKRS groups. **(D)** The negative correlation between the NKRS and immune score. **(E, F)** The positive correlation between the NKRS and tumor purity. **(G, H)** Heat maps illustrate the correlation between NKRGs and TIC levels calculated by the ImmunecellAI **(G)** and CIBERSORT **(H)** algorithms (*P<0.05, **P<0.01, ***P<0.001).

Pathway enrichment has led us to focus on tumor immunity and TME, so we evaluated the correlation between NKRS and TME using multiple algorithms: ImmunecellAI, CIBERSORT, TIMER, and ESTIMATE. The results indicated that NKRS was closely associated with several types of TICs like B cells, T cells, macrophages, and NK cells (**Supplementary Figure S3C**). Subsequently, we analyzed the TME differences between low-NKRS and high-NKRS groups. We observed a higher fraction of CD4^+^ T cells, CD8^+^ T cells, cytotoxic T lymphocytes, and NK cells in the low-NKRS group (**Figures 7A, B**). Moreover, the low-NKRS group also exhibited significantly fewer infiltrative M0 and M2 Macrophages, neutrophils, and more M1 Macrophages (**Figure 7B**). In addition, both the ImmunecellAI and CIBERSORT algorithms consistently demonstrated a strong correlation between NKRS and CD8^+^ cells and NK cells (**Figures 7A, B**). The violin plot showed that the low-NKRS group owned higher stromal, immune, and ESTIMATE scores, suggesting that NKRS was negatively correlated with the abundance of various TICs (**Figures 7C, D**). In addition, the high-NKRS group exhibited a higher tumor purity, further corroborating the relationship between NKRS and TME score (**Figures 7E, F**). The above results illustrated the significantly increased levels of anti-tumor immune cell components in the TME of the low NKRS group.

Furthermore, we generated heat maps to investigate the association between the expression of 7 signature NKRGs and TME (**Figures 7G, H**). Firstly, all seven NKRGs had a positive correlation with CD4^+^ T cells, CD8^+^ T cells, cytotoxic T lymphocytes, and NK cells. In contrast, the expression of these NKRGs negatively correlated with the abundance of neutrophils, monocytes, M0 macrophages, M2 macrophages, and Th17 (**Figure 7G**). Specifically, CCND2 demonstrated a strong correlation with the infiltration of effector T cells. Moreover, CCL5, IRF1, and KLRB1 share similar qualities, exhibiting a significantly positive correlation with CD8^+^ T cells, CD4^+^ T cells, cytotoxic T cells, NK cells, and M1 macrophages, as well as a negative correlation with macrophages M0 and M2 (**Figure 7H**). It was highlighted that CCL5, CCND2, and KLRB1 were the most pivotal factors, each exerting a significant influence on several crucial immune cell types. Thus, the seven NKRGs were intimately associated with various functional TICs within the TME.

### 3.9 Low-NKRS patients presented enhanced anti-tumor immunity and increased ICB sensitivity

**Figure 8:**
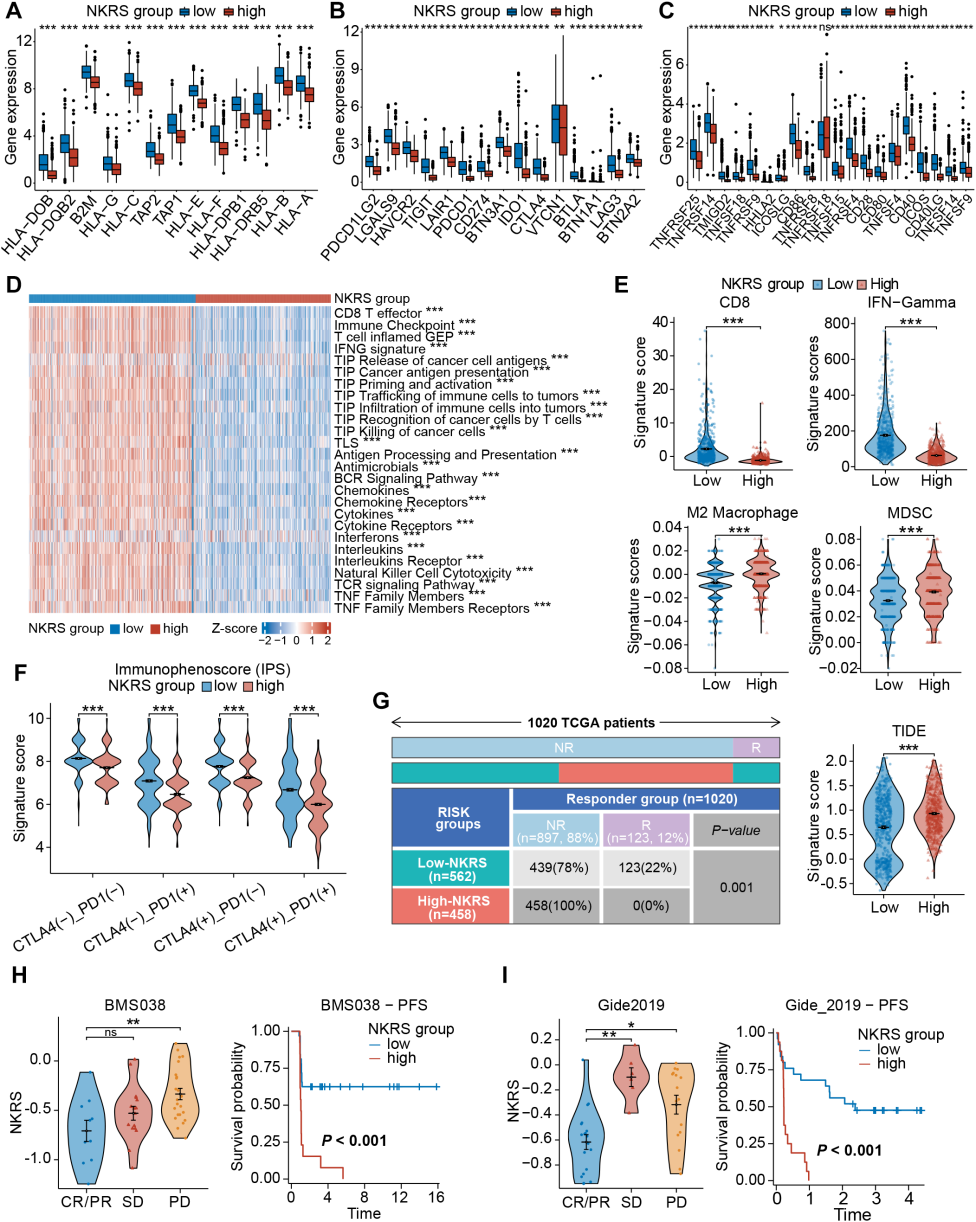
Evaluation of the immune function and immunotherapy sensitivity. **(A–C)** Box plots demonstrated significant differences in gene expression patterns related to immune function. **(D)** The correlation between NKRS and anti-tumor immune activities. **(E)** Violin plots revealed significant disparities in signature scores of CD8^+^ T cells, IFN-Gamma, M2 Macrophages, and MDSCs between the low- and high-NKRS groups. **(F)** Differences in IPS scores between different NKRS groups. **(G)** The distribution of responders and non-responders to immunotherapy and TIDE scores. **(H, I)** Validating the association between the NKRS and immunotherapy response in two ICB cohorts, BMS038 and Gide2019 cohorts (ns: not significant, *P<0.05, **P<0.01, ***P<0.001).

As the association between NKRS and TME has been confirmed, we hypothesized that NKRS would exert a similar effect on immune functional phenotypes. Differential expressions of genes related to immune function were discovered, with antigen processing- and presentation-related genes, as well as immune checkpoint genes, all being upregulated in the low-NKRS group (**Figures 8A-C**), confirming our suspicion. Some typical clinically used immune checkpoints were also up-regulated in the low-NKRS group, such as PD-L1, PDCD1, CTLA4, LAG-3, and CD86.

Subsequently, GSVA was performed to assess anti-tumor-related immunofunctional activities. Low-NKRS tumors exhibited significantly higher scores of the cancer-immunity cycle pathways, from the release of cancer cell antigens to cancer antigen presentation and, ultimately, the killing of cancer cells (**Figure 8D**). In addition, interferon, cytokine, and NK cell-mediated cytotoxicity activities were also upregulated in the low-NKRS group (**Figure 8D**), further illustrating that the low-NKRS group possesses a more robust immune response in combating BRCA. Moreover, GSVA based on other reference gene sets also revealed functional scores for CD8^+^ T cells, cytolytic activity, NK cells, and type I and type II IFN response were elevated in the low-NKRS group (**Supplementary Figure S4A**). These results illustrated the strong negative association between NKRS and anti-cancer immunity.

We further investigated the relationship between the NKRS and immunotherapy responsiveness. Calculated by the TIDE algorithm, the signature scores of CD8^+^ T cells and IFN-Gamma, major effectors in facilitating tumor killing, were higher in the low-NKRS group (**Figure 8E**). On the contrary, M2 macrophages and myeloid-derived suppressor cells (MDSCs), those mainly engaged in limiting anti-tumor immunity, scored higher in the high-NKRS group (**Figure 8E**). Consistently, the high-NKRS group also possessed higher TIDE scores, indicating the underlying poorer ICB efficacy of high-NKRS patients (**Figure 8G**). In terms of the immunophenoscore (IPS), the low-NKRS group had higher IPS scores regardless of the expression pattern of PD-1 or CTLA-4 (**Figure 8F**), further supporting our hypothesis.

Unsurprisingly, the proportion of participants responsive to ICB therapy was as high as 22% (123/562) in the low-NKRS group, whereas there were no responders (0%, 0/458) at all in the high-NKRS group, strongly endorsing the favorable predictive power of NKRS for immunotherapy responsiveness (**Figure 8G**). Several real-world patient cohorts receiving ICB therapy were utilized to test the predictive reliability of the NKRS. As observed in BMS038, Gide2019, and IMvigor210 cohorts, with the decreasing of NKRS, tumor growth became more manageable, resulting in a longer progression-free survival (PFS) and overall survival (OS) (**Figures 8H, I**; **Supplementary Figure S4B**). The above results suggested that NKRS is of promising value in predicting cancer immunotherapy efficacy, with lower NKRS correlating with better therapeutic benefits.

### 3.10 Low-NKRS patients may experience better chemotherapy outcomes

**Figure 9:**
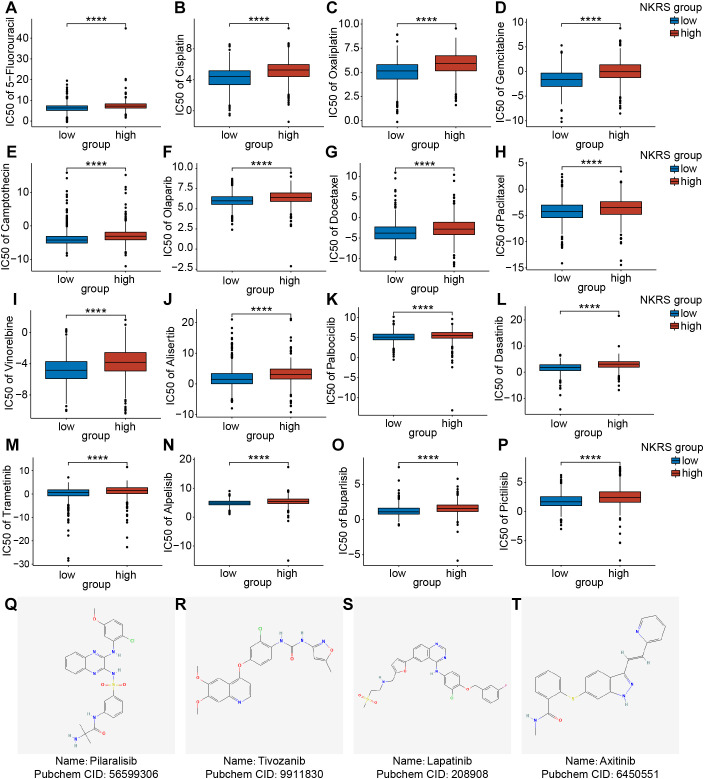
Assessment of the chemotherapeutic sensitivity and prediction of promising agents. **(A-P)** The IC50 values of 16 chemotherapeutic agents were analyzed and compared between the low- and high-NKRS groups. **(Q-T)** Utilizing the Cmap platform, 4 promising agents were identified for high-NKRS patients (****P<0.0001).

Chemotherapy is also a common non-surgical treatment for BRCA. We employed the “Oncopredict” package to investigate whether the NKRS could stratify patient populations with different chemotherapy susceptibilities. The IC50 values of drugs in the high-NKRS group were generally higher, indicating that high-NKRS individuals responded less favorably to most chemotherapeutic drugs, including DNA-targeting agents (5-Fluorouracil, Cisplatin, Oxaliplatin, Gemcitabine, Camptothecin, and Olaparib) (**Figures 9A-F**), cell cycle blockers (Docetaxel, Paclitaxel, Vinorelbine, Alisertib, and Palbociclib) (**Figures 9G-K**), and kinase inhibitors (Dasatinib, Trametinib, Alpelisib, Buparlisib and Pictilisib) (**Figures 9L-P**). Yet these results also illustrated the challenging issue of limited effective drugs in high-NKRS populations, so we identified 4 promising agents (Pilaralisib, Tivozanib, Lapatinib, and Axitinib) that might be suitable for high-NKRS patients using the Cmap, and their chemical structures were shown in **Figures 9Q-T**. These results demonstrated that NKRS was a useful indicator for guiding the clinical management of BRCA.

### 3.11 Randomized forest screening combined with experimental validation identifies KLRB1 and CCND2 as key prognostic NKRGs

**Figure 10:**
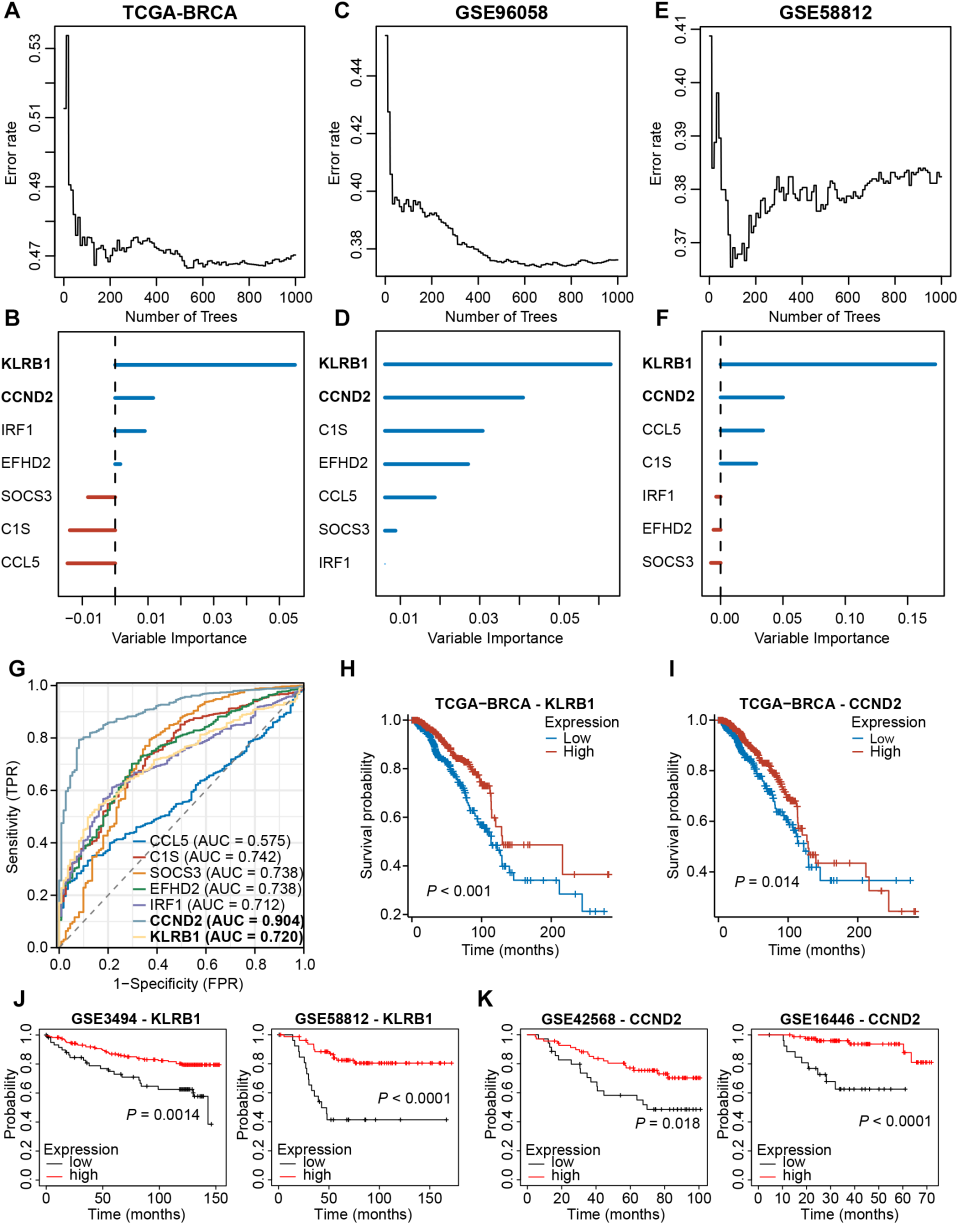
Screening for key NKRGs of importance to BRCA prognosis. **(A-F)** The machine learning method identified KLRB1 and CCND2 as key NKRGs in three cohorts. **(G)** Time-dependent ROC curves assessed the diagnostic accuracy of signature NKRGs. **(H, I)** Survival curves of BRCA patients with different KLRB1 or CCND2 levels in the TCGA-BRCA cohort. **(J)** Survival curves of BRCA patients with different KLRB1 levels in validation cohorts. **(K)** Survival curves of BRCA patients with different CCND2 levels in validation cohorts.

We then analyzed the expression profiles of these seven signature NKRGs, and we observed that the expression of three NKRGs (CCL5, EFHD2, IRF1) was up-regulated and the expression of four genes was down-regulated (KLRB1, C1S, SOCS3, CCND2) in BRCA tissues, with this trend being consistent across all the samples and paired samples (**Supplementary Figures S5A, B**). Aiming to further screen out important NKRGs with targeting value in this model, we incorporated the methodology of machine learning and screened for key NKRGs using the Random Forest algorithm. After analyzing the TCGA-BRCA dataset, KLRB1 and CCND2 were identified as important NKRGs due to their highest importance on patient prognosis (**Figures 10A, B**). More importantly, we got a consistent finding in two external cohorts, verifying the importance of KLRB1 and CCND2 (**Figures 10C-F**). Moreover, ROC curves showed the reliable performance of these 7 NKRGs, with AUC of 0.720 and 0.904 at KLRB1 and CCND2, respectively, representing a high reliability (**Figure 10G**). Furthermore, in both the TCGA-BRCA cohort and multiple validation cohorts, higher expression of KLRB1 or CCND2 was capable of predicting longer OS of BRCA patients (**Figures 10H-K**). The aforementioned results unequivocally demonstrated that KLRB1 and CCND2 are crucial prognostic NKRGs in BRCA and possess significant clinical value for future applications.

Further assessment of the clinicopathological relevance of the key genes revealed that the expression of both KLRB1 and CCND2 was correlated with the pathological stage of the tumor and the age of the patients, with no significant correlation with the N-stage (**Supplementary Figures S6A, C, E**). There was a certain negative association between CCND2 and the T-stage (**Supplementary Figure S6B**). KLRB1 was closely related to the survival status and the PAM50 subtypes (**Supplementary Figures S6F, G**). Although the expression of KLRB1 was lower in metastatic tumors, the difference was not statistically significant (**Supplementary Figure S6D**).

**Figure 11:**
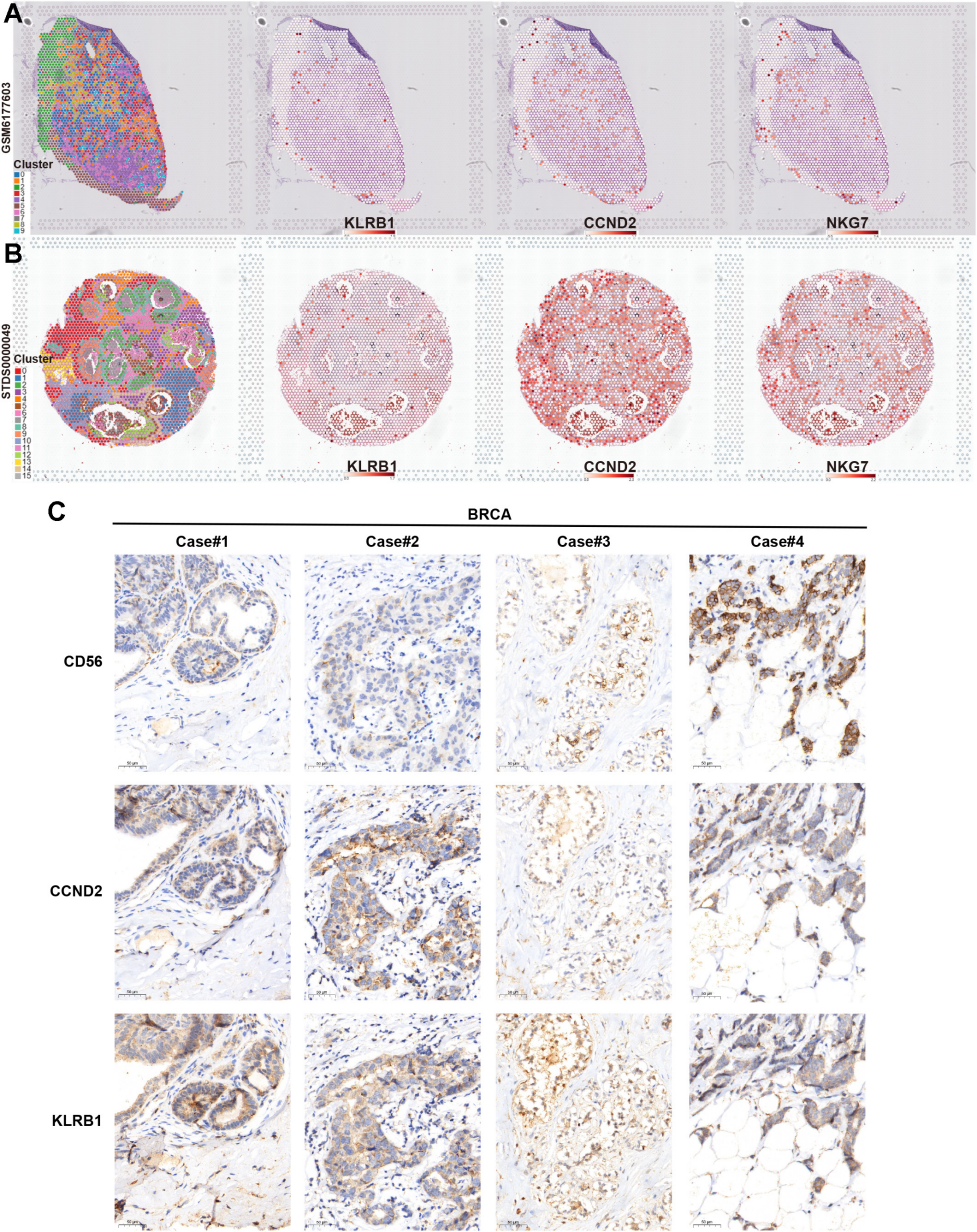
Verifying the association between two key NKRGs and NK cells using spatial transcriptome and IHC assays. **(A, B)** Spatial transcriptomic slides confirmed the positive correlation between the abundance of KLRB1 and CCND2 and the NK cell marker NKG7 in BRCA tissues. **(C)** Tissue microarray-based IHC assays validated the positive correlation between KLRB1 and CCND2 and the NK cell marker CD56 in BRCA specimens.

Finally, we verified the association between key NKRGs, KLRB1 and CCND2, and tumor-infiltrating NK cells by combining spatial transcriptomics and IHC assays based on BRCA tissue specimens. Two publicly available spatial transcriptomic sections of BRCA revealed a strong positive correlation between the spatial expression of KLRB1 and CCND2 and the NK cell marker NKG7 (**Figures 11A, B**). Further IHC staining using BRCA tissue microarrays showed that both KLRB1 and CCND2 showed a certain spatial co-localization with CD56, a typical marker of NK cells (**Figure 11C**). These results strongly suggested the prognostic significance and immune microenvironmental relevance of key NKRGs, KLRB1 and CCND2.

## 4 Discussion

Breast cancer (BRCA) presents a significant global health challenge and profoundly affects women’s well-being (2, 3). Consequently, it is crucial to develop personalized, risk-based screening strategies for early detection and effective therapeutic management of BRCA. The association between prognosis and immune cells within the tumor microenvironment (TME) has been extensively validated across various cancer types (50–52). While current research primarily focuses on adaptive immune cells, including B cells, CD4^+^ T cells, and CD8^+^ T cells, the role of innate immune cells remains underexplored and underreported (51, 53, 54). Notably, only a subset of patients respond favorably to immunotherapies targeting adaptive immune cells. Therefore, developing therapeutic prediction models based on innate immune-related molecules is a promising research direction.

The efficacy of immunotherapy is influenced by various factors within the tumor microenvironment (TME). Thus far, strategies that target innate immunity have demonstrated significant promise in clinical cancer research (55). Key components in TME, such as dendritic cells, CD8^+^ T cells, and macrophages, play important roles in cancer immunity. NK cells, in particular, demonstrate potent anti-tumor activity through the recognition and elimination of malignant cells (56–58). Activated NK cells can eradicate tumor cells by releasing perforin and granzyme or by inducing apoptosis via ADCC, FasL, or TRAIL pathways (59, 60). Moreover, NK cells can secrete cytokines, incorporating IFN-γ and TNF-α, which inhibit tumor growth (61). They also facilitate the recruitment of dendritic cells into the TME (62). Emerging evidence suggests that a novel subset of NK cells can even attract T cells into the TME, enhancing the effectiveness of ICB therapy (63). In recent years, the pivotal role of NK cells in tumorigenesis has been increasingly acknowledged. Though several studies have employed NKRGs as reliable biomarkers to predict immunotherapy response rates in cancers such as lung and gastric cancer, there is a notable lack of relevant studies in the field of BRCA (64–66). Inspired by these studies, along with the realistic demands, we aim to investigate the prognostic and molecular characteristics of tumor-infiltrating NK cells in BRCA and identify novel NK-related molecular biomarkers. Our goal is to develop an NKRS model to enhance the prognosis assessment and advance precision medicine in BRCA.

In the present study, we observed a significantly poorer survival rate among BRCA samples with lower contents of NK cells compared to those with higher contents, thereby establishing a correlation between NK cells and BRCA prognosis. We also used a novel screening strategy for key genes, combining the single-cell transcriptome and the bulk transcriptome-based WGCNA, resulting in 49 candidate NKRGs. Following the LASSO regression, we established a seven-gene (CCL5, EFHD2, KLRB1, C1S, SOCS3, IRF1, and CCND2) NK-related prognostic and therapeutic prediction signature, serving as an independent and reliable prognostic indicator for BRCA.

Among these 7 NKRGs comprising the signature, some have been reported to significantly influence cancer progression, while others remain understudied. For instance, existing research indicates that increased SOCS3 promotes the degradation of IDO, an immunosuppressive molecule secreted by MDSCs (67). Furthermore, SOCS3 overexpression considered as a potential therapeutic target for TNBC and hepatocellular carcinoma, aligning with our finding (68). C1S, a key gene in the classical complement pathway, acts as an adjunct component that can potentiate antibody-based immunotherapies (69). CCND2, a cyclin known for inducing a senescent phenotype and inhibiting cell growth, observed deletions or mutations in many BRCA samples (70). In addition, CCND2 expression was found to increase during cell growth arrest, suggesting its suppressing role in cancer development (71). EFHD2 has been reported to exhibit immunomodulatory and inflammatory regulatory functions in non-alcoholic steatohepatitis (NASH) (72), but its involvement in tumors remains unexplored. Several NKRGs, like CCL5, demonstrate dual effects on tumors by promoting both tumor progression and enhancing anti-tumor immune responses (73). Specifically, CCL5 promotes the infiltration of regulatory T cells (Tregs) into the TME, thereby favoring tumor growth (74). However, CCL5 also facilitates the infiltration of CD8^+^ T cells to kill tumor cells (75). Regarding KLRB1, our findings align with those of Jiu-Ling Chen and other researchers, who discovered that KLRB1 was a tumor suppressor gene suggestive of a better prognosis, and that KLRB1 expression was positively correlated with the level of CTLs, B cells and DCs (76, 77). Conversely, other studies have also indicated that KLRB1 may diminish T cell-mediated cytotoxicity and induce NK cell dysfunction, contributing to tumor progression (78, 79). The reason for this contradiction may be the cellular heterogeneity of KLRB1 expression, whose functions in tumor and immune cells may differ. In addition, since KLRB1 is mainly expressed in NK cells and T cells, there is a possibility that its high expression suggesting a better prognosis may also result from a higher level of infiltrative immune effector cells such as NK and T cells. Thus, KLRB1 emerges as a pivotal immunomodulatory molecule, intricately regulating the delicate balance of NK cell and T cell activity. The transcription factor IRF1 is involved in the regulation of PD-L1 expression (80). It also specifically binds to the promoters of immunosuppressive genes in tumor cells, thereby hindering the anti-tumor immune response (81). Also, IRF1 is associated with the activation of dendritic cells (82). Among these 7 NKRGs, we used machine learning combined with multi-cohort analyses to identify the significant contribution of KLRB1 and CCND2 to BRCA prognosis and validated their correlation with NK cells by spatial transcriptomics and specimen IHC assays. More subsequent studies are warranted to further elucidate the functional and prognostic significance of these NKRGs.

Given the robust prognostic effect of NKRS in BRCA, we applied the GSEA algorithm and discovered a remarkable enrichment of the chemokine signaling pathway, natural killer cell-mediated cytotoxicity pathway, T-cell receptor signaling pathway, and inflammatory response pathway in the low-NKRS group. These are well-established pathways that foster anti-tumor immune responses (83–86). So, we analyzed the correlation between NKRS and TME. The results revealed a strong association between NKRS and NK cells, T cells, and other immune cell types. We also found almost all NKRGs involved in this signature positively correlated with CD4^+^ T cells, CD8^+^ T cells, cytotoxic T lymphocytes, and NK cells. These cells are capable of directly or indirectly eliminating tumor cells across numerous cancer types (84, 85, 87, 88). Given the established negative correlation between NKRS and the infiltration level of various effector immune cells, we observed a similar effect on immune functional phenotypes. Several typical immune checkpoints currently in clinical use, including PD-1, CTLA-4, and PD-L1, were also observed to be upregulated in the low-NKRS group, suggesting that these patients may exhibit an enhanced response to ICB therapies (89). Although we observed higher immune checkpoint expression in the low-NKRS group, and it is commonly believed that upregulated checkpoints such as PD-L1 are associated with immune evasion and immunosuppression, we also observed improved immune cell infiltration, enhanced antigen-presenting, enhanced effects of effector cytokines such as interferon (IFN) and tumor necrosis factor (TNF), and enhanced immune recognition and killing in the low-NKRS group, and therefore the low upregulation of immune checkpoints in the NKRS group may be due to a combination of several of these anti-tumor microenvironmental factors. Indeed, we corroborated the association between a lower NKRS and a better ICB responsiveness with comprehensive validations, including immune function scores, TIDE and IPS algorithms, and survival and differential analysis based on real-world ICB cohorts.

Despite the fact that high-NKRS BRCA patients presented lower sensitivity to immunotherapy and most chemotherapeutic agents in this study, we identified four effective agents (Pictilisib, Tivozanib, Lapatinib, and Axitinib) that might be suitable for them. Pictilisib is a type I PI3K inhibitor that induces apoptosis and inhibits the proliferation of centroblasts and tumor cells (90). Tivozanib, an inhibitor of the vascular endothelial growth factor receptor (VEGFR), impedes tumor angiogenesis, thereby limiting tumor growth and blood flow. Tivozanib is primarily used in treating advanced renal cell carcinoma, where it has demonstrated significant efficacy in prolonging progression-free and overall survival (91). Lapatinib is a dual tyrosine kinase inhibitor that can inhibit the proliferation and activity of tumor cells by simultaneously acting on the epidermal growth factor receptor (EGFR) and human epidermal growth factor receptor 2 (HER-2). For patients with advanced HER2^+^ BRCA, Lapatinib has demonstrated significant therapeutic efficacy (92). Axitinib, a second-generation selective inhibitor of VEGFR, is primarily utilized for treating renal cell carcinoma (RCC), particularly in cases where immunotherapy has failed (93). Further clinical trials are necessary to explore and validate the application of these chemotherapeutic agents in BRCA.

In summary, through the integration of single-cell and bulk transcriptomic analyses, we identified 7 NKRGs associated with ICB resistance, which were further employed to establish an NK-related risk score (NKRS). This NKRS exhibited robust predictive capabilities in various aspects, encompassing patient survival, TME landscape, immune functions, immunotherapy response, and chemotherapy sensitivity. In addition, we applied machine learning methods to identify 2 key NKRGs, with their prognostic roles and TME relevance also validated by multi-cohort analysis, spatial transcriptomics, and specimen-based IHC experiments.

Nevertheless, this study has several limitations. Firstly, the effects of the 7 NKRGs were solely confirmed through database analysis, necessitating further exploration of their specific functions and molecular mechanisms. Additionally, our analysis was confined to NK cells in the TME, overlooking a diverse array of other cells and cytokines, so the model was rendered less comprehensive. Ultimately, our study provides a novel NK cell-based perspective for the prognostic assessment and individualized treatment of BRCA patients, with potential contributions to precision medicine and the identification of new therapeutic targets for BRCA.

## Data Availability

The original contributions presented in the study are included in the article/**Supplementary Material**. Further inquiries can be directed to the corresponding authors.

## Supplementary material

Supplementary Figure 1 Additional analysis results from scRNA-seq and WGCNA. **(A)** Feature plots revealed the differential expression of KLRD1 among cell clusters. **(B)** Violin plots demonstrate that KLRD1 was predominantly expressed in NK cells. **(C)** Preliminary gene clustering in WGCNA. **(D)** Preliminary clustering of gene modules and merging minor modules.

Supplementary Figure 2 Validation of the clinical value of NKRS in external cohorts. **(A)** Confirming the NKRS was an independent prognostic indicator in three validation cohorts. **(B)** The histograms and box plots further supported the clinical relevance of NKRS in validation cohorts (*P<0.05, ***P<0.001, ****P<0.0001).

Supplementary Figure 3 Additional GSEA and immune infiltration results. **(A, B)** GSEA results based on HALLMARK gene sets in the high- and low-NKRS groups. **(C)** The correlation between NKRS and immune cell abundance calculated by different algorithms.

Supplementary Figure 4 Analysis of immune function and immunotherapy. **(A)** GSVA analysis revealed differences in immune function between NKRS groups. **(B, C)** The violin plot and survival analysis on the IMvigor210 cohort validated the capability of NKRS in predicting ICB efficacy (***P<0.001).

Supplementary Figure 5 Expression profiles of signature NKRGs in BRCA. **(A)** Expression patterns of signature NKRGs in BRCA and non-malignant tissues. **(B)** Expression patterns of signature NKRGs in BRCA and paired paracancerous tissues (*P<0.05, ***P<0.001).

Supplementary Figure 6 Clinical relevance of key genes KLRB1 and CCND2. Association between the expression of key NKRGs and pathologic stage **(A)**, TNM stage **(B-D)**, patient age **(E)**, survival status **(F)**, and PAM50 subtypes **(G)** (*P<0.05, **P<0.01, ***P<0.001).
