# Supplementary figures and images for: Comprehensive single-cell and bulk transcriptomic analyses to develop an NK cell-derived gene signature for prognostic assessment and precision medicine in breast cancer
Source: Front Immunol. 2024 Oct 23;15:1460607. doi: 10.3389/fimmu.2024.1460607 (PMC11537931; doi:10.3389/fimmu.2024.1460607)

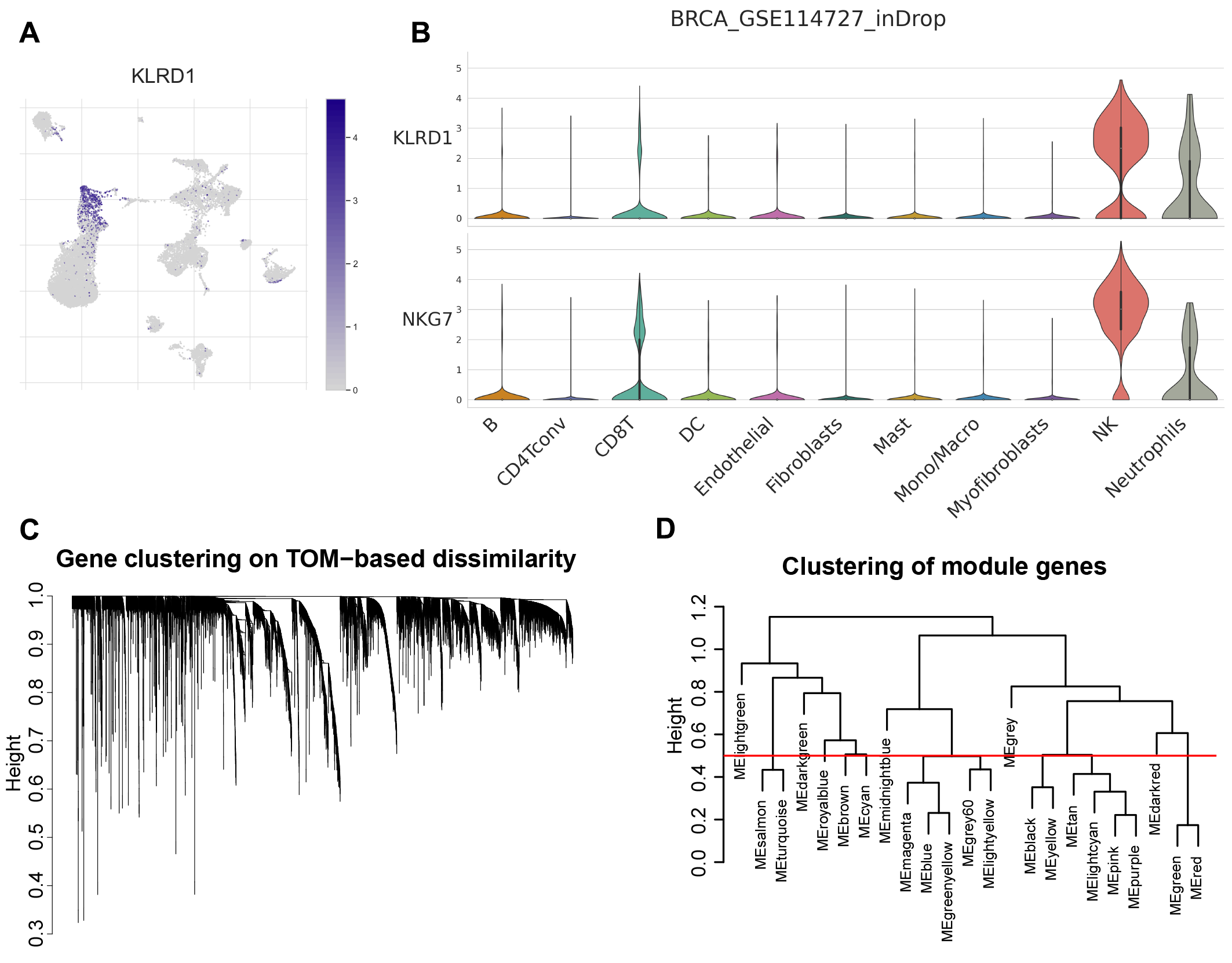

Supplement: Supplementary Figure 1 — Additional analysis results from scRNA-seq and WGCNA. (A) Feature plots revealed the differential expression of KLRD1 among cell clusters. (B) Violin plots demonstrate that KLRD1 was predominantly expressed in NK cells. (C) Preliminary gene clustering in WGCNA. (D) Preliminary clustering of gene modules and merging minor modules. [file DataSheet1.zip › Figure S1.tif]

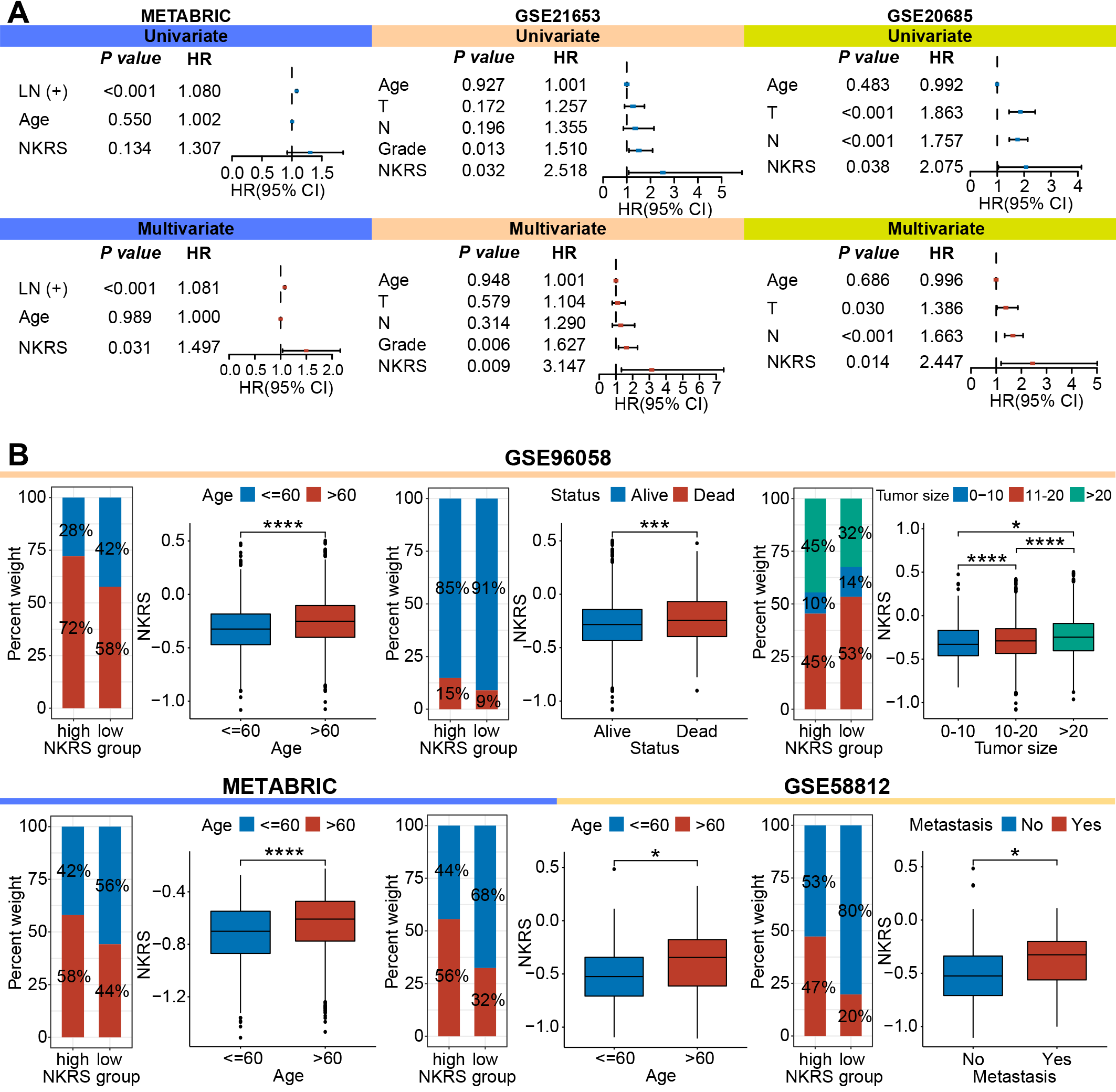

Supplement: Supplementary Figure 1 — Additional analysis results from scRNA-seq and WGCNA. (A) Feature plots revealed the differential expression of KLRD1 among cell clusters. (B) Violin plots demonstrate that KLRD1 was predominantly expressed in NK cells. (C) Preliminary gene clustering in WGCNA. (D) Preliminary clustering of gene modules and merging minor modules. [file DataSheet1.zip › Figure S2.tif]

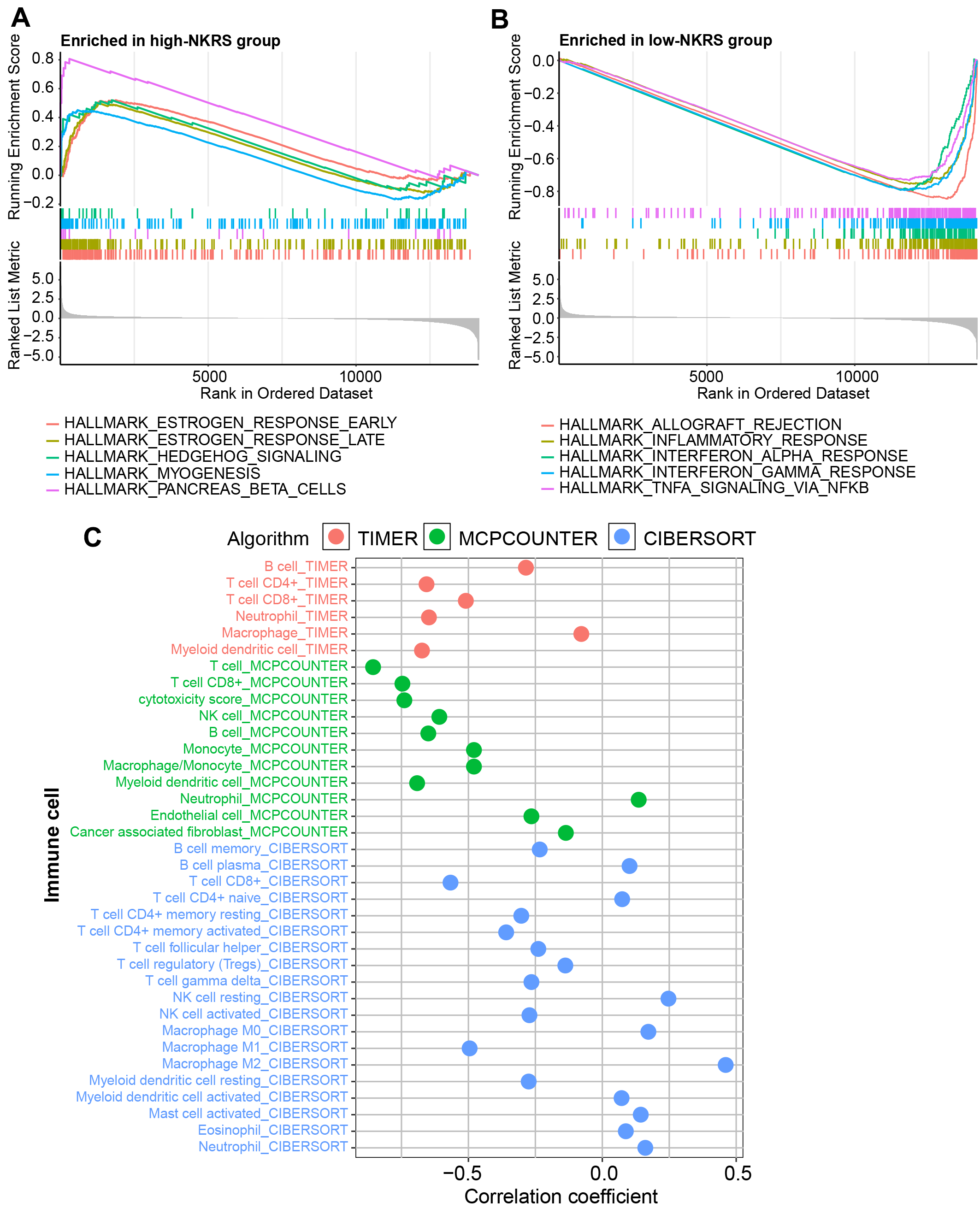

Supplement: Supplementary Figure 1 — Additional analysis results from scRNA-seq and WGCNA. (A) Feature plots revealed the differential expression of KLRD1 among cell clusters. (B) Violin plots demonstrate that KLRD1 was predominantly expressed in NK cells. (C) Preliminary gene clustering in WGCNA. (D) Preliminary clustering of gene modules and merging minor modules. [file DataSheet1.zip › Figure S3.tif]

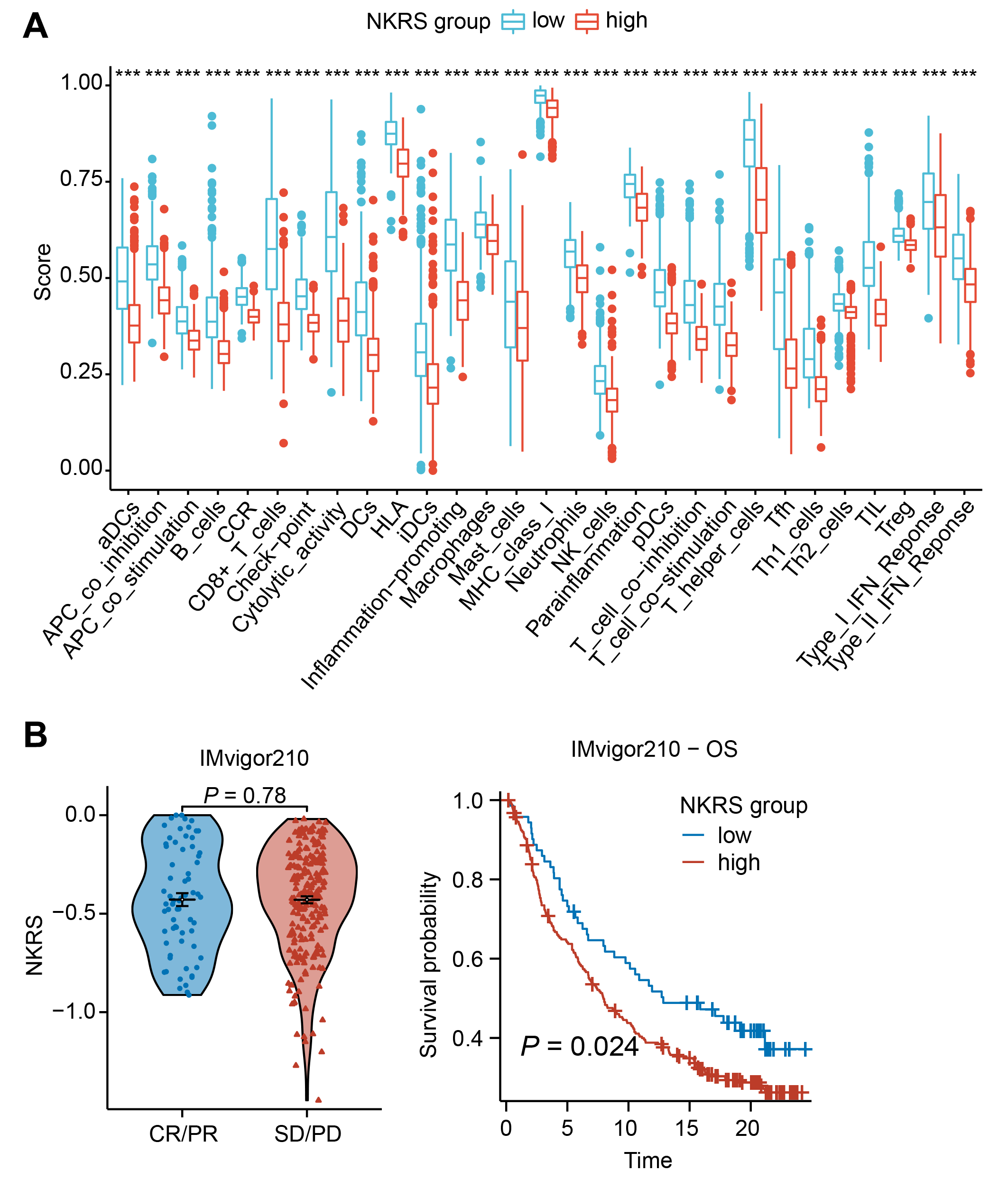

Supplement: Supplementary Figure 1 — Additional analysis results from scRNA-seq and WGCNA. (A) Feature plots revealed the differential expression of KLRD1 among cell clusters. (B) Violin plots demonstrate that KLRD1 was predominantly expressed in NK cells. (C) Preliminary gene clustering in WGCNA. (D) Preliminary clustering of gene modules and merging minor modules. [file DataSheet1.zip › Figure S4.tif]

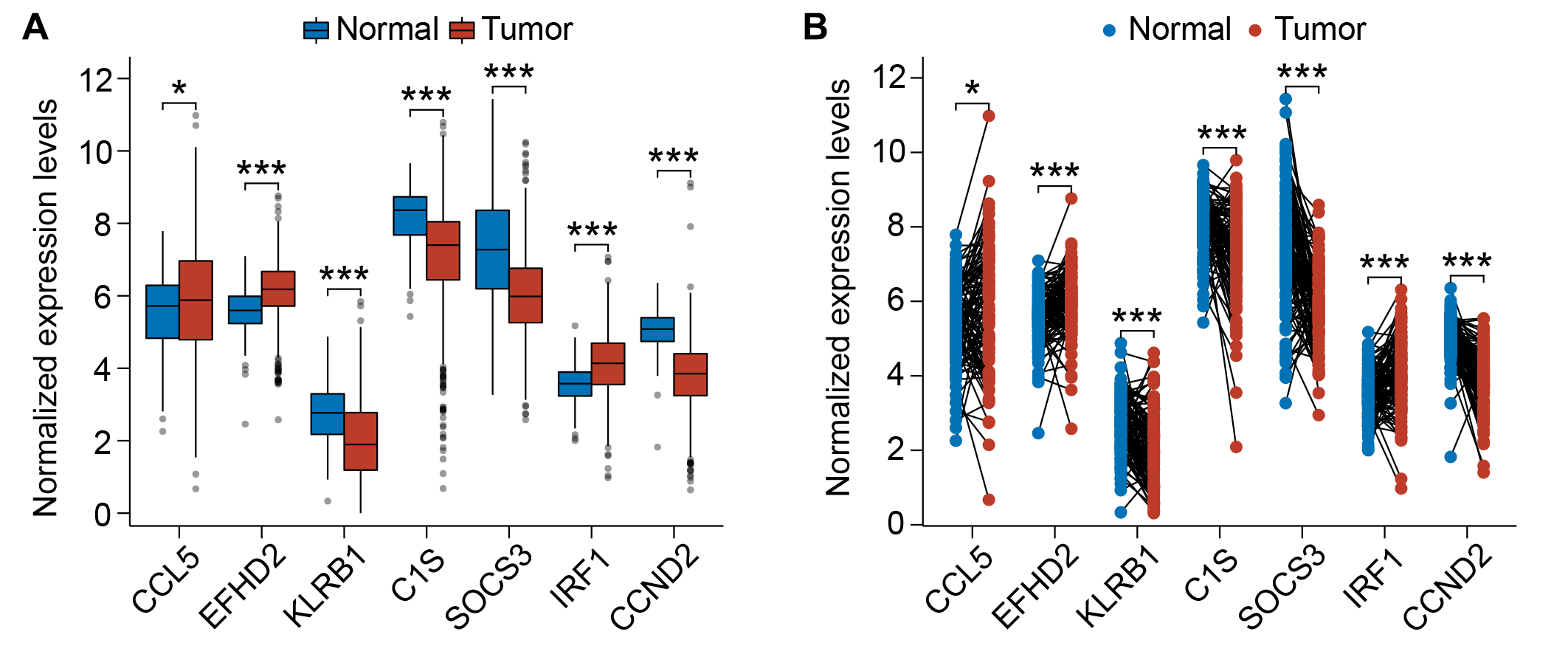

Supplement: Supplementary Figure 1 — Additional analysis results from scRNA-seq and WGCNA. (A) Feature plots revealed the differential expression of KLRD1 among cell clusters. (B) Violin plots demonstrate that KLRD1 was predominantly expressed in NK cells. (C) Preliminary gene clustering in WGCNA. (D) Preliminary clustering of gene modules and merging minor modules. [file DataSheet1.zip › Figure S5.tif]

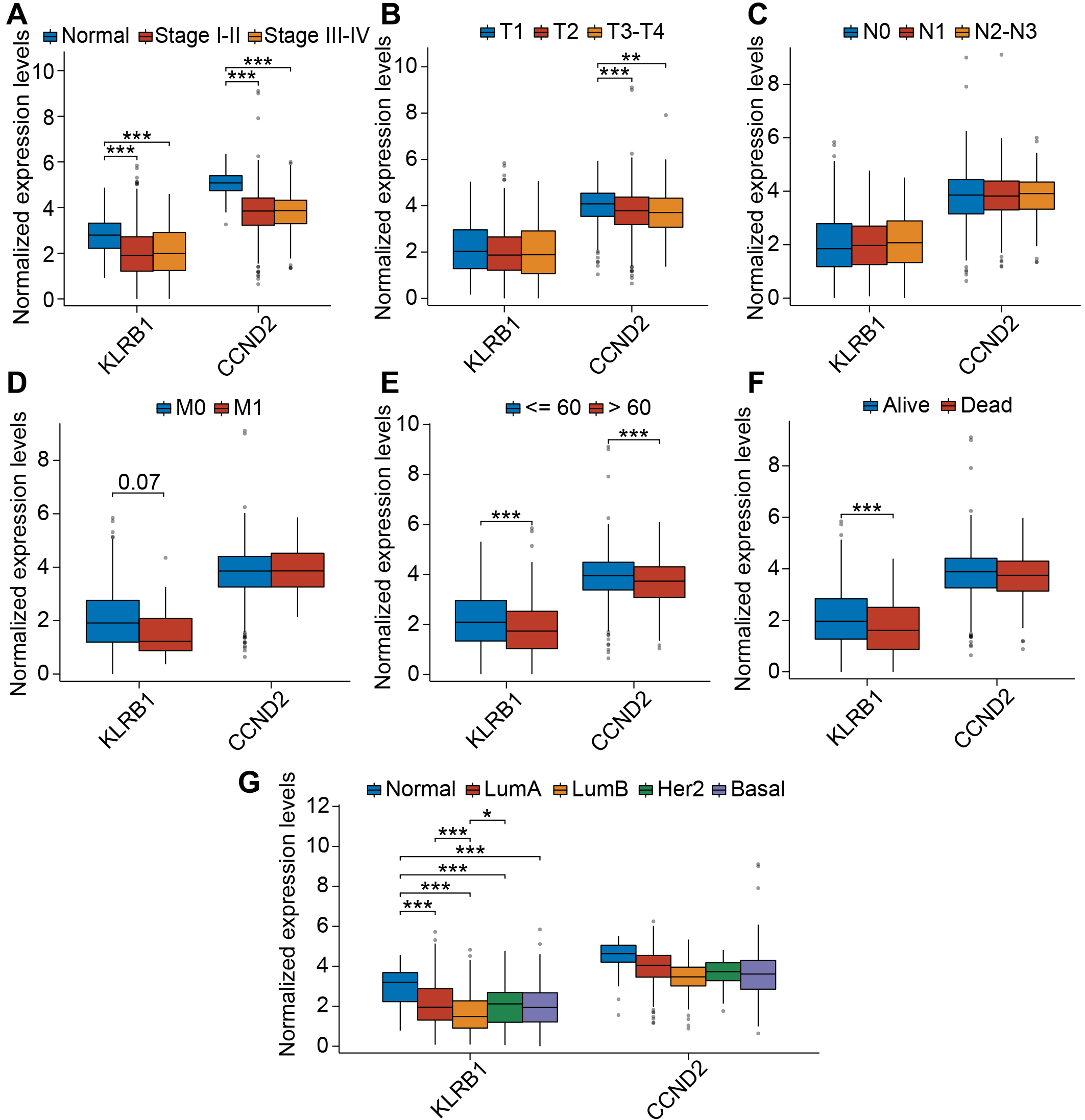

Supplement: Supplementary Figure 1 — Additional analysis results from scRNA-seq and WGCNA. (A) Feature plots revealed the differential expression of KLRD1 among cell clusters. (B) Violin plots demonstrate that KLRD1 was predominantly expressed in NK cells. (C) Preliminary gene clustering in WGCNA. (D) Preliminary clustering of gene modules and merging minor modules. [file DataSheet1.zip › Figure S6.tif]
